# Transmission of social bias through observational learning

**DOI:** 10.1126/sciadv.adk2030

**Published:** 2024-06-28

**Authors:** David T. Schultner, Björn R. Lindström, Mina Cikara, David M. Amodio

**Affiliations:** ^1^ Faculty of Social and Behavioral Sciences, Department of Psychology, University of Amsterdam, Amsterdam, Netherlands.; ^2^ Division of Psychology, Department of Clinical Neuroscience, Karolinska Institutet, Stockholm, Sweden.; ^3^ Graduate School of Arts and Sciences, Department of Psychology, Harvard University, Cambridge, MA, USA.

## Abstract

People often rely on social learning—learning by observing others’ actions and outcomes—to form preferences in advance of their own direct experiences. Although typically adaptive, we investigated whether social learning may also contribute to the formation and spread of prejudice. In six experiments (*n* = 1550), we demonstrate that by merely observing interactions between a prejudiced actor and social group members, observers acquired the prejudices of the actor. Moreover, observers were unaware of the actors’ bias, misattributing their acquired group preferences to the behavior of group members, despite identical behavior between groups. Computational modeling revealed that this effect was due to value shaping, whereby one’s preferences are shaped by another’s actions toward a target, in addition to the target’s reward feedback. These findings identify social learning as a potent mechanism of prejudice formation that operates implicitly and supports the transmission of intergroup bias.

## Teaser

Viewing a prejudiced person’s intergroup behavior induces that bias in observers, revealing a mechanism of bias propagation.

## INTRODUCTION

The ability to learn from the experiences of others is fundamental to human survival (*1*). By observing another person’s choices and outcomes, we are better positioned to gain rewards and avoid harms when facing those choices ourselves (*2*, *3*). Social learning—learning through the observation of others—informs processes ranging from fear learning to complex moral judgments (*4*–*12*), and it has been proposed as a crucial mechanism for the transmission of cultural knowledge across individuals (*1*). Here, we propose that social learning also contributes to the transmission of human intergroup prejudice. We asked: when observing an actor’s interactions with members of a social group, does the observer acquire the group-based preferences of the actor? And what is the learning mechanism? By addressing these questions, we sought to illuminate a process through which prejudices may spread between individuals and contribute to societal-level inequality.

Research on bias contagion suggests that one’s intergroup attitudes can be influenced by observing others’ intergroup interactions (*13*). When participants observed clips of interracial interactions on TV shows, an actor’s body language toward a group member was found to communicate group-based preferences to the observer, which subtly influenced the observer’s own racial attitude (*14*–*16*). In other research, children who viewed an actor’s displays of positive or negative nonverbals toward a target person adopted the same preferences as their own (*17*, *18*).

Bias contagion findings are consistent with the possibility that prejudice can be transmitted between individuals through observational learning. To date, however, research has not directly addressed the question of prejudice transmission—that is, whether an actor’s own degree of prejudice is acquired by the observer—nor the learning process through which this may occur. Moreover, while bias contagion studies have focused on the effect of viewing an actor’s expressive behavior toward a target, they have not examined the interplay of responses between an actor and target that characterize a social interaction.

In direct social interactions, involving a repeated exchange between two partners, an actor can form a partner impression through instrumental learning—that is, by acting toward the partner and learning from their feedback (*19*, *20*). Studies of interracial interaction show that a person’s prejudices tend to be expressed in their nonverbal behavior (*15*, *21*–*23*), which in turn can influence how people approach intergroup interactions and form preferences through instrumental learning in direct social interactions (*24*, *25*).

In observational instrumental learning, an observer views the choices of a demonstrator in a social interaction and learns from both the demonstrator’s actions and the feedback they receive from the target (*3*, *26*, *27*). Prior research on observational instrumental learning has focused on interactions with nonsocial targets, such as when an observer learns the reward value of different shapes by watching a demonstrator choose among them and receive feedback on their choices (*27*, *28*). However, individuals can also learn about other individuals by observing social interactions in which one person acts and the other responds. For example, a newly hired employee (observer) can learn about her new colleagues by observing whom a fellow worker (demonstrator) approaches for help and whether that person (target) offers it.

A key difference between observational and direct forms of reinforcement learning concerns the sources of reinforcement. In direct reinforcement learning, one learns from the reward feedback of a chosen target (*29*). By contrast, in observational learning, one can learn from two sources: (i) the demonstrator’s action and (ii) feedback from the chosen target (*28*, *30*, *31*). Each source of reinforcement may produce a separate prediction error (i.e., discrepancy between expectancy and outcome) such that an observer may update their preferences through an action prediction error, based on demonstrator choice, as well as a reward prediction error, based on target feedback (*27*).

The simultaneous availability of these two sources of reinforcement may create a unique kind of ambiguity: when learning from a demonstrator’s actions, it may be unclear to the observer whether a choice reflects the demonstrator’s preference or a characteristic of the target. Consider, for example, a manager who likes one employee more than another, despite his mediocre performance, and tends to choose him for work events. An observer could misinterpret such choices as indicating the employee’s competence rather than the manager’s preference—a misattribution that would lead the observer to form positive impressions of the employee despite his middling performance.

This process of misattribution in observational learning corresponds to a “value shaping” mechanism in computational models of social reinforcement learning. According to value shaping, a demonstrator’s choice frequency for an option directly shapes an observer’s preference for that option, resulting in more favorable impressions of options that were chosen more often by the demonstrator (*28*). This process suggests a simple computational mechanism for how misattribution might emerge from a combination of observed actions and rewards to produce a bias in social learning.

Can value shaping lead to the formation of prejudice in observers who view an intergroup interaction? In intergroup observational learning, an observer views a demonstrator’s choice to interact with a group member and learns from both the demonstrator’s choice and the group member’s feedback. If there is ambiguity about the reason for a choice, the observer may misattribute a demonstrator’s personal preference to an attribute of the group member (i.e., value shaping). To the extent that an observer acquires a group-level preference from individual-level observations, despite no actual group differences, it would represent the formation of prejudice (*32*, *33*).

This misattribution process in observational learning suggests an implicit mode of prejudice transmission: It assumes that observers are unaware that their impressions are influenced by the demonstrator’s actions and instead attribute them to genuine group differences—an indirect (i.e., implicit) effect of demonstrator choice on an observer’s group perceptions (*34*). Consequently, the observer may have little reason to correct this bias in their own interactions with group members (*35*). Together, these effects suggest an unexplored mechanism through which prejudice may be acquired and transmitted via the observation of intergroup interactions.

In the present research, we investigated this observational instrumental learning process of prejudice transmission. In six studies (total *N* = 1550), participants observed interactions between a person with prior stereotype knowledge (demonstrator) and members of stereotyped groups (targets). We hypothesized that observers of these interactions would acquire the preferences expressed by the demonstrator, despite being naïve to the stereotypes driving demonstrators’ preferences. We used computational modeling to disentangle the respective contributions of demonstrator actions and target rewards on an observer’s own choice behaviors. Furthermore, we propose a misattribution account for this effect, whereby observers misattribute a demonstrator’s choice preferences to a group member’s reward value (i.e., value shaping), and that this learning generalizes from individuals to their groups, consistent with the formation of prejudice.

## RESULTS

### Study 1

In study 1, each participant observed previous interactions between a demonstrator and members of two social groups, taken from a prior study of direct social-interactive learning (*24*). In this prior study, participants (demonstrators) were exposed to positive and negative stereotype descriptions regarding two social groups, respectively. Participants were led to believe that players were real people from real social groups, and group descriptions were based on stereotypes of White and Black Americans [full stereotype descriptions can be found in the Supplementary Materials; (*36*)]. Participants were told that, to maintain the anonymity of the groups and players, groups would be referred to only as “Group B” and “Group G” (for “Blue” and “Green,” respectively), and players would be represented by avatars they had chosen. Participants then interacted with members of each group in a social reinforcement learning task, presented as a money sharing game, in which they could choose and learn from members of each group. Although this task lacked many features of a natural social interaction, it involved the essential components of social instrumental learning: repeated rounds of choice and feedback between two individuals.

**Fig. 1.**
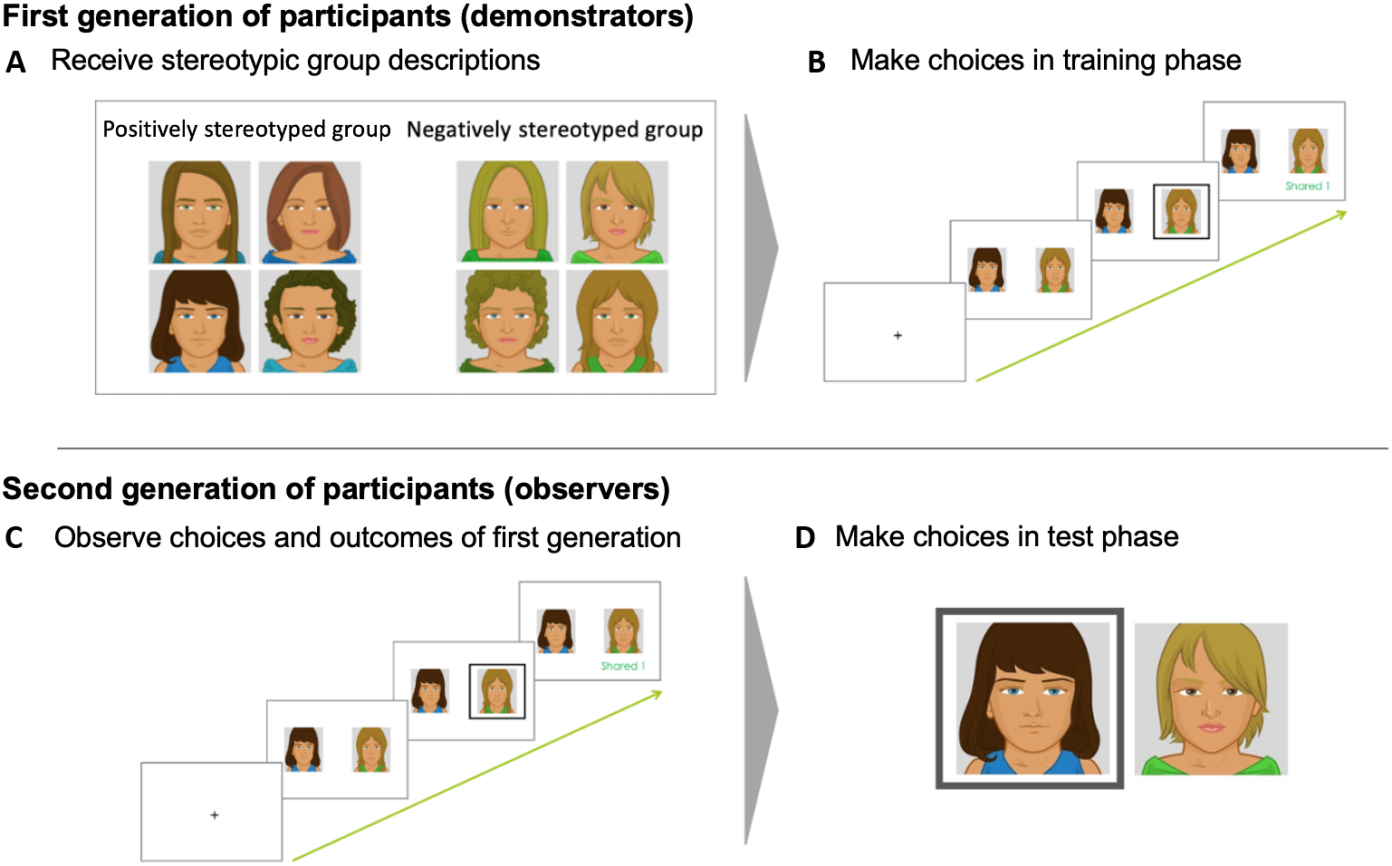
Schematic of study design. (**A**) A first generation of participants (demonstrators) viewed group stereotype descriptions. (**B**) Demonstrators then made choices between group members and received feedback in a training phase. (**C**) A second generation of participants (observers) observed the training phase choices and reward feedback of one yoked first-generation demonstrator. (**D**) Observers then made their own incentivized test phase choices.

Training phase choices from this earlier study provided interaction stimuli for the present study. On each of 160 training phase trials, the demonstrator was presented with pictures of two players, one from each group, and chose one of these players to interact with (Fig. 1). The chosen player then responded with reward or nonreward monetary feedback. Although individual players varied in their sharing rate, reward probabilities were equated between groups. Nonetheless, choice behaviors of these prior participants (demonstrators), on average, showed a preference for players from the positively stereotyped group in a subsequent test phase (β = 0.52, SE = 0.06, Wald *z* = 9.33, *P* < 0.001), in addition to a preference for individuals with higher sharing rates (*27*).

In study 1 of the present research, each of a new sample of 290 university undergraduate laboratory-based participants (observers; 54% female) viewed the full set of training phase interactions between a demonstrator and group members. On each trial, they viewed the demonstrator’s choice followed by reward feedback from the chosen player. Each demonstrator session was viewed by either two or three observers in a yoked fashion. Although individual players varied in their feedback, sharing rates between groups were equated by design; moreover, despite variability in feedback revealed by idiosyncratic demonstrator choices, realized sharing rates did not differ by group across participants (*t* = 0.52, df = 45079, *P* = 0.61). After viewing the demonstrators’ choices in a learning phase, participants made their own choices in a test phase with the same players, this time viewing all possible pairings of members from each group and choosing the player expected to share to win a cash bonus (96 trials). Feedback was not displayed during the test phase to prevent new direct learning.

**Fig. 2.**
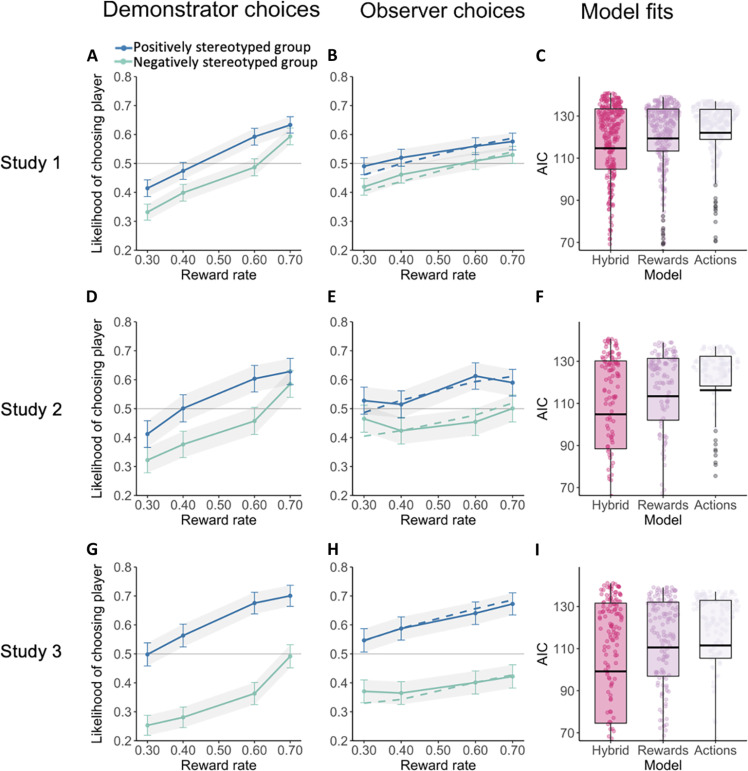
Choice behavior and model fits. (**A**, **D**, and **G**) Demonstrator choice behavior in the learning phase in studies 1 to 3. Demonstrators showed both a reward effect (slope of lines) and a group bias (distance between lines). Error bars indicate SEM. (**B**, **E**, and **H**) Observers in studies 1 to 3 showed a matching reward and group effect in their own test phase choices. Solid lines indicate choice behavior, and dashed lines indicate predictions from the best-fitting hybrid model. (**C**, **F**, and **I**) Comparison of model fits from reward learning, action learning, and hybrid families for studies 1 to 3. Dots indicate individual participants’ model fit, bold horizontal lines indicate mean AIC values, and box plots indicate 50% interquartile ranges.

We first asked whether observers acquired the group bias expressed by the demonstrator, in addition to learning individual players’ actual reward rates. A logistic mixed-effects regression revealed both effects: While observers’ choices reflected actual sharing rates of individual players (β = 1.15, SE = 0.16, Wald *z* = 7.14, *P* < 0.001), they also reflected a unique effect of social group, consistent with the demonstrators’ average group preference (β = 0.30, SE = 0.12, Wald *z* = 2.63, *P* = 0.008; Fig. 2B). Hence, despite equated sharing rates between groups, observers exhibited the group bias expressed by their demonstrators. Moreover, the degree of an observer’s acquired group bias (i.e., proportion of choices favoring members of the positively stereotyped group) reflected the magnitude of their respective demonstrator’s group preference (β = 0.41, SE = 0.08, Wald *z* = 5.10, *P* < 0.001), corresponding to a correlation of 0.29 (*t* = 5.10, df = 288, *P* < 0.001).

These results show that participants learned from both the group members’ feedback and the demonstrator’s choices, suggesting that observers integrated both sources of information into their subjective valuations of target groups. To test this hypothesis directly, we fit participants’ choice behavior to computational models in which behavior is explained by either (i) observed reward outcomes (*29*), (ii) observed actions (*27*, *28*), or (iii) a combination of observed reward outcomes and actions (hybrid models; see the Supplementary Materials for full description of modeling approach). Model fits were compared using the Akaike information criterion (AIC), a goodness-of-fit measure that penalizes models with additional parameters to control for overfitting. Model comparison indicated that a hybrid model with parameters for both demonstrator’s actions and target’s reward feedback (with separate learning rates for positive and negative prediction errors) provided the best fit to behavioral data, corroborating the multilevel regression results (Fig. 2C). Comparison of the reward- and action-based learning rate effects revealed that, on average, the impact of target feedback was twice as large as the impact of demonstrator actions (see table S3). Hence, observers formed their preferences based on the combination of demonstrator actions and group member feedback, consistent with a value shaping account.

Next, we tested whether observers misattributed demonstrators’ choice preference to the sharing rates of targets. To this end, participants were asked to report the sharing rate of each player [“What percent of the time did this player share a point? (0-100)”]. Although the average player sharing rate was equated between groups, observers reported higher sharing rates from the group preferred by the demonstrator (β = 3.50, SE = 1.43, *t* = 2.40, *P* = 0.015). This misperception aligned closely with demonstrators’ choices: observers’ perception of sharing differences between players was associated with the actual choice bias expressed by the demonstrator (β = 21.66, SE = 10.4, *t* = 2.08, *P* = 0.038; Fig. 2C) and also by the action learning parameter derived from the hybrid model (β = 3.36, SE = 1.56, *t* = 2.16, *P* = 0.032). These results directly support a misattribution account rooted in value shaping.

To test whether this transmission of group preference was implicit—that is, whether participants were unaware of the demonstrator’s influence on their preferences—we asked participants to report their knowledge of demonstrators’ choice tendencies (“What percent of the time did the actor you observed choose this player?”; from 0 to 100% of choices). Although observers correctly identified demonstrators’ preference for higher sharing players across groups (reward effect: β = 31.29, SE = 1.83, Wald *z* = 17.10, *P* < 0.001), they failed to notice the demonstrators’ group preference [group effect: β = 1.70, SE = 1.45, Wald *z* = 1.17, *P* = 0.24, Bayes factor (BF, incl) = 0.047], similar to (*17*). Crucially, observers’ choice preferences were more strongly predicted by their (mis)perceptions of player sharing rates than by their perceptions of demonstrator preferences (*F* = 21.7, *P* < 0.001, linear contrast of standardized β coefficients), indicating that the demonstrator’s bias was implicitly misattributed to the behavior of group members.

Finally, we tested whether observers’ learned preferences generalized from individual players to a group-level representation (i.e., prejudice). That is, when observing a demonstrator’s interactions, did the observer merely acquire preferences for individual group members or for their social group as well? To address this question, we compared two versions of the best-fitting model: one in which action learning was specified as occurring at the individual level, and the other at the group level. This analysis indicated that observers acquired group-level preferences from individual-level observations (57% group-level versus 43% individual-level Akaike weights, difference using a *t* test: *t* = 4.68, df = 578, *P* < 0.001; see full modeling results in the Supplementary Materials). Thus, observational learning from demonstrator interactions with individual group members resulted in group-level representation, consistent with the formation of a prejudice (*32*).

Together, these results demonstrate the observational learning of prejudice: Observers acquired the group preferences expressed by demonstrators despite equivalent reward feedback from members of each group. Furthermore, observers were unable to report demonstrators’ preferences and instead misattributed them to group members’ reward feedback. Computational models confirmed that both demonstrator choices as well as target reward feedback guided their own group-based preferences, consistent with a value shaping mechanism.

### Study 2

Study 2 (*N* = 114, 39% female) repeated the procedure of study 1 with a nonstudent online sample (workers on Amazon Mechanical Turk). As in study 1, observers acquired the group preferences of demonstrators (β = 0.83, SE = 0.35, Wald *z* = 2.41, *P* = 0.016; Fig. 2E), in addition to learning from player’s actual reward feedback (β = 1.12, SE = 0.25, Wald *z* = 4.5, *P* < 0.001), and observers’ choice data were fit best by the computational hybrid model (see the Supplementary Materials; Fig. 2F). Participants again misperceived a group difference in player feedback (β = 3.7, SE = 1.2, Wald *z* = 3.10, *P* = 0.0019), and their degree of misperception was associated with the degree of group preference expressed by the demonstrator whom they observed (β = 38.07, SE = 11.26, Wald *z* = 3.38, *P* < 0.001). These results replicated the main empirical findings of study 1, demonstrating the observational learning of prejudice and supporting a misattribution account.

### Study 3

While the results of studies 1 and 2 demonstrated a relationship between demonstrator bias and observers’ learned preferences, the demonstrators in those studies varied widely in their choice bias, with many exhibiting no bias or preferences running counter to the stereotype descriptions they viewed (average group preference in study 1: 53.8%, SD = 12.73%, study 2: 55%, SD = 14.82%). To more directly demonstrate an effect of demonstrator bias on observer preferences, study 3 participants (*N* = 140, 39% female) completed the same task as in studies 1 and 2 but viewed interactions involving demonstrators who exhibited above-median group bias (average preference: 63.4%, SD = 12.06%; Fig. 2G). This would also potentially make it easier for observers to explicitly perceive demonstrators’ bias and subsequently correct for it in their own choice behavior. Nevertheless, results indicated a clear group bias in observers’ choices (β = 1.45, SE = 0.31, Wald *z* = 4.69, *P* < 0.001; Fig. 2H), providing a more direct demonstration of observational prejudice formation. Moreover, observers’ group bias in study 3 (group preference: 61%) was stronger than that of either study 1 or study 2 (53% in both studies, *t* = 3.84, df = 249, *P* < 0.001). Yet, despite the pronounced expression of preference by demonstrators in study 3, observers continued to mistakenly perceive a group difference in players’ sharing rates (β = 6.60, SE = 1.00, Wald *z* = 6.40, *P* < 0.001; perceived demonstrator preference was not measured in study 3).

Together, studies 1 to 3 show that group-based preferences, which originated from stereotype messages communicated directly to demonstrators in a prior session, were propagated to novel participants through observational instrumental learning. Furthermore, this transmission was due to the observer’s misattribution of the demonstrator’s preference to the value of the target player—an effect rooted in a computational value shaping mechanism.

Next, studies 4 to 6 were conducted to further probe the proposed misattribution account, first by addressing potential alternative explanations and then by investigating the extent to which the observational learning effect relies on attributions of human demonstrator preferences.

### Study 4

**Fig. 3.**
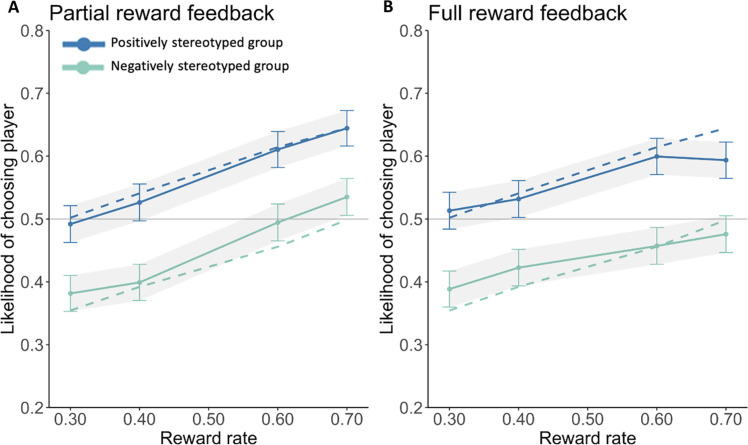
Observers’ test phase choice behavior under partial or full reward feedback. (**A**) Observers’ choice behavior under partial reward feedback in study 4. (**B**) Observers’ choice behavior under full reward feedback. Solid lines indicate choice behavior, and dashed lines indicate model predictions.

Prior research shows that prejudice may form through the biased sampling of information from group members: If a learner samples only from a preferred group, they would miss potential positive experiences with another group (*37*, *38*). The observation of biased sampling behaviors would also presumably skew one’s group preferences. To determine whether our results were due to biased sampling, in study 4 (*N* = 339, 40% female), we manipulated whether observers viewed the feedback of chosen players only, as in studies 1 to 3, or the feedback of both the chosen and unchosen player, displayed simultaneously on each trial. As in study 3, participants viewed choices of high-bias demonstrators. Results showed that access to complete reward information did not reduce the observer’s expression of group preferences; that is, the group learning effect was not moderated by feedback condition [Group × Feedback condition interaction: β = 0.13, SE = 0.34, Wald *z* = 0.38, *P* = 0.70, BF(incl) = 0.07, strongly supporting the absence of an interaction effect; Fig. 3]. Separate analyses within each condition showed that observers formed significant group-based preferences in response to partial feedback (β = 0.67, SE = 0.24, Wald *z* = 2.82, *P* = 0.004) and to full feedback (β = 0.86, SE = 0.23, Wald *z* = 3.69, *P* < 0.001). Moreover, the correlation (*r* = 0.17, *t* = 3.16, df = 337, *P* = 0.002) between demonstrators’ and observers’ group preferences did not differ between conditions (β = −0.16, SE = 0.21, *t* = −0.77, *P* = 0.40).

These results rule out a biased sampling account for the transmission of group preferences through social learning; biases were propagated at similar rates despite observers’ access to full reward information of both social groups, strengthening support for the value shaping account of demonstrators’ actions. Although it is possible that participants attended more to feedback from chosen targets, the fact that demonstrators sampled frequently from each group (47% versus 53%) further contradicted a biased sampling explanation.

### Study 5

Next, we asked whether the observational learning of prejudice effect depends on a human demonstrator. According to the misattribution account, observers acquire prejudice because they assume that the demonstrator’s choice reflects the target’s value. One possibility is that this effect involves a mental state inference regarding the demonstrator’s beliefs, and thus only occurs when a demonstrator is human. However, it is also possible that mental state inference is not necessary, and that merely observing the choice of group members is sufficient to induce inferences of their value.

We tested these alternatives in study 5. Participants (*N* = 364, 42% female) were assigned to view interactions involving either a human demonstrator or the “randomly determined selections” of a computer demonstrator. Target players in both conditions were presented as human participants. Additionally, participants in both conditions viewed the learning phase behavior of high-prejudice demonstrators, as in study 3; this design was intended to produce a large group-based learning effect in the human condition, seen in study 3, against which any decrease in the computer condition could be more powerfully detected. Following the learning task, participants self-reported their perceptions of demonstrator selections and targets’ reward rates.

Results showed that observers’ choice preference again reflected the group-based preference of the demonstrator (β = 1.14, SE = 0.23, Wald *z* = 4.98, *P* < 0.001), in addition to players’ actual reward feedback (β = 1.32, SE = 0.16, Wald *z* = 8.29, *P* < 0.001). Crucially, this group-based preference was not moderated by condition (β = 0.60, SE = 0.33, Wald *z* = 1.84, *P* = 0.067), emerging for both the human (β = 1.12, SE = 0.22, Wald *z* = 5.10, *P* < 0.001) and computer (β = 1.77, SE = 0.25, Wald *z* = 7.23, *P* < 0.001) demonstrators. These results suggest that the observational learning bias did not depend on a human demonstrator, but that merely viewing a bias in selection frequency was sufficient.

Self-report data showed that while observers perceived the group-based preference of these high-prejudice demonstrators (β = 8.32, SE = 1.58, *t* = 5.25, *P* < 0.001), they also misperceived a group difference in players’ rewards (β = 22.41, SE = 2.34, *t* = 9.59, *P* < 0.001). Across conditions, this misperception of player feedback more strongly predicted their own choice preferences, relative to their perception of demonstrator preferences (*F* = 49.65, *P* < 0.001; see the Supplementary Materials).

Together, the results suggest that a human demonstrator is not necessary for the social learning of prejudice, and thus, the effect may not require mental state inference of the demonstrator. Nevertheless, observers again misperceived the value of group member targets from the choices of the demonstrator, human or nonhuman, and formed their own biased impressions of group members based on this misattribution.

### Study 6

Although mental state inferences may not be necessary for the observational learning of prejudice, as in the case of a computer demonstrator, it remains possible that mental state inference drives this effect when observing human demonstrators. In study 6 (*N* = 303, 51% female), we manipulated participants’ beliefs about the demonstrator’s competence while holding all other aspects of the design constant. If a demonstrator’s choices are inferred to reflect their informed decisions about a target, then observational learning should be stronger when the demonstrator is viewed as more competent (*39*).

Before viewing a demonstrator’s interactions with group members, participants learned that the demonstrator performed either above average (high competence condition) or below average (low competence condition) on an ostensible prior reasoning task—a manipulation adapted from past social learning experiments (*40*). A manipulation check assessing post-task ratings of demonstrator competence confirmed that the demonstrator was perceived as more competent in the high-competence (*M* = 69.87, SD = 16.49) than the low-competence (*M* = 45.17, SD = 18.83) condition (*t* = −12.64, df = 323, *P* < 0.001). Participants in each condition then viewed demonstrator interactions, randomly drawn from the same set used in study 1, and completed their own test phase choices.

**Fig. 4.**
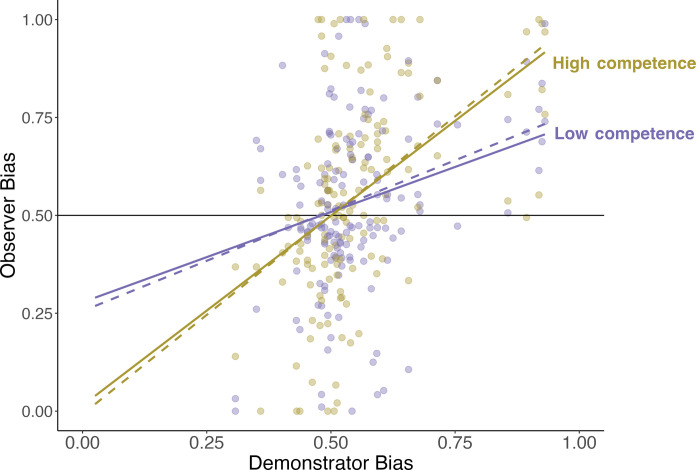
In study 6, perceived demonstrator competence moderated the association between demonstrator bias and observer bias (solid lines) such that observed demonstrator biases were acquired more strongly if their source was depicted as competent. Model-derived predictions, based on simulations from the reward/action hybrid model (dotted lines), captured this pattern. The interaction between competence and demonstrator bias remained significant when excluding extreme values of demonstrator bias (see the Supplementary Materials).

As predicted, observers’ group preferences were shaped more strongly by demonstrators framed as competent (equivalent to Spearman ρ = 0.51, *S* = 319437, *P* < 0.001) than as incompetent (equivalent to Spearman ρ = 0.30, *S* = 554260, *P* < 0.001; interaction using robust regression: β = −0.42, SE = 0.19, Wald *z* = −2.35, *P* = 0.018; Fig. 4). Despite the stronger correspondence between demonstrators’ and observers’ preferences in the high-competence condition, it is notable that individual demonstrators varied widely in their degree and direction of group bias, and thus, the average magnitude of the group bias did not differ between conditions [Group membership × Demonstrator competence interaction: β = −0.24, SE = 0.38, Wald *z* = −0.62, *P* = 0.53, BF(incl) = 0.01, strongly supporting the absence of a condition moderator]. These results show that observers’ learning was influenced by their perception of demonstrator competence, suggesting that a mental state inference regarding a demonstrator’s knowledge can enhance the biasing effect of group-based social learning.

## DISCUSSION

Despite the crucial role of social learning in adaptive decision making, we show that it can also contribute to the transmission of prejudice between individuals. Across six experiments, we demonstrated that group preferences can be acquired by merely observing the behavior of a prejudiced actor toward members of a group. This effect emerged despite observers’ lack of stereotype knowledge, unawareness of demonstrator preferences, the lack of actual group differences in players’ feedback, and the use of financial incentives for accuracy, suggesting that observational learning constitutes a potent and persistent mode of prejudice transmission.

Studies 1 to 3 demonstrated the observational learning of prejudice: Participants who observed a demonstrator’s choice preferences toward group members later expressed a similar degree of preference in their own behavior. Study 1 further showed that participants attributed their group-based preferences to the behavior of players, whose feedback was equated between groups, more than to the biased preferences of the demonstrator. Study 2 replicated the observational learning effect, and study 3 showed that viewing the behavior of a high-prejudiced demonstrator produced similarly high levels of prejudice in the observer. Computational modeling confirmed that observers assigned reward value to the demonstrator’s actions in addition to learning from the sharing behavior of target group members, and that they integrated these representations when producing their own choice preferences.

Studies 4 to 6 refined our understanding of the value shaping mechanism underlying observational learning. Study 4 showed that the observational learning of prejudice persisted when observers viewed feedback from both the chosen and unchosen group members on each trial, suggesting that the effect was not likely due to a sampling bias (*38*, *41*). Study 5 revealed that this social learning bias did not require the demonstrator to be human; observers inferred the value of group members based on their selection by a computer demonstrator. However, study 6 showed that beliefs about demonstrator competence enhanced the biased learning effect, indicating a role for mental state inference when learning from interactions involving human demonstrators.

Together, these studies show that an observer can acquire the group-based preferences of a demonstrator by merely viewing their behaviors toward group members. This form of prejudice formation occurred without the observers’ prior stereotype knowledge or awareness of the demonstrators’ preference; rather, observers attributed their preferences to (mis)perceived differences in the group members’ behavior. Finally, although observers sought to learn the reward values of individual group members, their individual-level preferences generalized to the group, consistent with the formation of a group-based prejudice.

Our findings consistently support a misattribution account of observational learning of prejudice. According to this model, an observer misattributes the choice preferences of a demonstrator to the behavior of group members. Study 1 participants did not detect the real bias in demonstrators’ choices, yet they perceived a (nonexistent) difference in the sharing rate between members of the two groups—a misperception that guided their own choice preferences.

The findings of study 6 suggest that this misattribution effect involves a mental state inference of the demonstrator: Observers assume a demonstrator’s choice reflects knowledge of a target and thus attribute the choice not to the demonstrator’s personal preference but to the value of the target. Notably, however, misattribution also occurred for a computerized demonstrator in study 5; we speculate that this misattribution was due either to participants’ anthropomorphization of the computer (*42*, *43*), consistent with a mental state inference account, or to greater attentional processing of selected targets, shown previously to increase valuation (*44*). Thus, while our findings broadly support the role of mental state inference in this misattribution effect, other mechanisms (e.g., differential attention) may also contribute when observing interactions involving a nonhuman demonstrator, such as a robot or artificial intelligence (AI).

An implication of this account is that prejudices formed through misattribution may be highly persistent. If an observer is unaware of the source of their preference and misattributes it to their direct veridical experience with a group member’s behavior, then they would have no reason to question or correct it (*19*). Furthermore, this prejudice could be transmitted to others in a self-perpetuating pattern: If an observational learner has subsequent direct interactions with group members, a new person may observe this interaction and form similar preferences, spreading it further across a community. This process of observational learning and propagation suggests an unexplored form of prejudice transmission, which, given its implicit operation, may require new proactive or systemic interventions for its mitigation (*45*–*47*).

The misattribution effect found here differs from previously studied forms of attribution, in which situational influences on a person’s behavior are misattributed to the person’s character (*48*–*50*). Here, observers misattributed the actions of one person (a demonstrator) to another (the group member). We suggest that this occurred in part because the bias in demonstrators’ choices was probabilistic and difficult to track explicitly. This previously unexplored form of misattribution, seen here in the context of observational learning, may offer a useful model for understanding the interindividual spread of prejudice while also suggesting an explanation for previous observations of bias contagion.

Using computational modeling to disentangle the contribution of observed actions and rewards, we showed that while observers learned from the target’s responses, they also incorporated the actor’s choices into their impressions. We propose that value shaping—the tendency to incorporate others’ choices into one’s own preferences—provides a mechanistic account for how demonstrator actions affect observers and produce a misattribution effect.

Whereas some previous computational models of group preference formation focused on selective sampling explanations (*38*, *51*), study 4 showed that group-based preferences can emerge even with full access to reward information. We speculate that in real-world intergroup contexts, which include myriad features excluded from the present experimental designs, effects of value shaping and biased sampling likely operate in concert to facilitate the transmission of prejudice (*52*). As such, the observation-based spread of prejudice may be reduced by alerting observers to the potential for bias in a demonstrator’s behavior, focusing observers’ attention on behaviors of a target person, or selectively exposing observers to unbiased or positive intergroup interactions [e.g., (*53*)].

A limitation of this work was its reliance on experimental tasks that, while permitting rigorous tests of our behavioral and computational hypotheses, presented only a minimal form of social interaction to observers. Nevertheless, our findings of prejudice transmission are consistent with those obtained in more ecologically valid designs (*14*, *17*), suggesting that the mechanisms identified here operate in more immersive social learning contexts.

More broadly, this research illuminates a pathway through which individual-level prejudice may spread to higher-level social structures such as communities and societies. We suggest that observational instrumental learning, whereby one person’s prejudice is transmitted to another through value shaping and misattribution, provides an important basis for this effect. This process likely interacts with social structures and stereotypes to perpetuate and maintain existing patterns of bias and inequality in real-world intergroup contexts. These findings thus raise new questions regarding the interplay of individual, dyadic, and systemic modes of prejudice formation, and while advancing our understanding of social learning, they pose new challenges for interventions aimed at prejudice reduction.

## MATERIALS AND METHODS

### Experimental design

#### Stimuli, task, and procedure

The task comprised two phases: an observational learning phase, in which participants observed another person’s interaction choices with members of two groups, and a test phase, in which participants made choices in the game for themselves. Before the observational learning phase, participants were told that they would learn about members of two social groups by observing a previous participant’s interactions with group members. Unbeknownst to observers, demonstrators had been exposed to positive and negative stereotype messages about the two groups, respectively, which induced their choice preference. In all experiments, group members were described as past participants who were represented by eight avatar images, either all male or all female (counterbalanced across participants). Unlike demonstrators, observers received no information regarding the players’ social groups.

#### Observational training phase

Demonstrators’ behavior was taken from Schultner *et al.* (*24*) and, after excluding missed trials, presented as completed by previous participants. The task was adapted from (*54*); demonstrators made binary choices between one member of each group and learned whether chosen targets shared a point with them. Participants viewed four different pairs of players, each with a member of the positively and negatively stereotyped groups. However, sharing rates of the two players differed across pairs (30% versus 70%, 40% versus 60%, 60 versus 40%, and 70% versus 30%, respectively) such that reward probabilities were equated between groups.

On each learning phase trial, two targets, one from each group, were presented, upon which the demonstrator chose to interact with one of them within 2000 ms. Reward feedback (“Shared: 1 point” or “Shared: 0 points”) appeared immediately following choice and was shown for 1500 ms. Each participant saw the entirety of one randomly selected previous participant’s task behavior. Counterbalancing and randomization occurred at the level of the demonstrator such that avatar gender, stimulus-to-reward mappings, trial order, and reward outcomes were randomized for the first generation of participants and then presented to second-generation participants in a fixed manner. Participants observed up to 160 learning trials, separated in two blocks. After a pseudo-random series of trials during the observational learning phase, participants completed 20 attention checks by indicating which of the two available targets was chosen on the previous trial.

#### Test phase

After a break screen, participants completed a test phase with up to 96 trials, depending on the number of trials the demonstrator completed, in which participants made binary choices between previously encountered targets. No reward feedback was provided. During the test phase, every possible between-group target pair combination was shown, in contrast to the fixed pairs shown in the training phase. This design assessed responses to both novel and previously viewed pairs, permitting a fine-grained measure of learned reward associations.

All tasks were completed online and were programmed using HTML, CSS, and JavaScript within the platform psiTurk. All experiments were hosted using a webserver at New York University.

### Participants

Participants in Exp. 1 (*n* = 359) were New York University undergraduates recruited from the psychology participant pool. Participants in Exps. 2 to 6 were recruited from Amazon Mechanical Turk: *n* = 152 in Exp. 2, *n* = 158 in Exp. 3, *n* = 387 in Exp. 4, *n* = 427 in Exp. 5, and *n* = 355 in Exp. 6. Participants in Exp. 1 received course credit and a monetary bonus of up to $2. Participants in Exps. 2 to 6 received $2 to $4.50 for their participation and up to $3 in bonus, depending on their test phase performance. Informed consent was obtained from all participants. Exp. 1 was approved by the New York University Institutional Review Board (IRB); Exps. 2, 3, 5, and 6 were approved by the University of Amsterdam IRB; and Exp. 4 was approved by the Harvard University IRB.

Participants who failed to respond correctly to at least 50% of catch trials were excluded from analysis. This criterion excluded 33 participants in Exp. 1, 43 participants in Exp. 2, 11 participants in Exp. 3, 31 participants in Exp. 4, 52 participants in Exp. 5, and 37 participants in Exp. 6. Moreover, data from trials with response times <200 ms were removed, and participants with fewer than 75% valid trials in the test phase (i.e., 72/96 trials) were excluded. Following exclusions, sample sizes for analysis were 290 (Exp. 1, 54% female, mean age 19.39 ± 1.26 years), 114 (Exp. 2, 39% female, mean age 33.54 ± 9.24 years), 140 (Exp. 3, 39% female, mean age 41.72 ± 12.42 years), 339 (Exp. 4, 40% female, mean age 40.69 ± 11.32 years), 364 (Exp. 5, 42% female, mean age 38.35 ± 11.42 years), and 303 (Exp. 6, 49% female, mean age 40.76 ± 12.25 years). Following a larger sample in Exp. 1, sample sizes were determined through power analyses with Cohen’s *d* = 0.3 for the group effect and 80% power, yielding at least *n* = 150 for single-condition studies (Exps. 2 and 3) and *n* = 300 for two-condition studies (Exps. 4 to 6). Final sample sizes deviated to some extent from target sample sizes due to inconsistent data quality and resulting exclusions.

### Statistical analyses

All statistical analyses were performed in R Studio [RStudio Team (2020). RStudio: Integrated Development for R. RStudio, PBC, Boston, MA; http://www.rstudio.com/]. Regressions were performed using the lme4 package [v1.1-26; (*55*)], and figures were made using the ggplot2 package (*56*). All statistical analyses were performed with maximal random effects structures, including random slopes for reward and group predictors, nested within participants, as well as random intercepts for participants. For Bayesian analyses, we performed Bayesian model comparison. We compared different regression models using the Bayesian information criterion (BIC) to BF method, in which a BF is computed by comparing the BIC of models including and excluding an additional predictor (*57*). This technique makes use of a unit information prior (UIP).

### Models

#### Reward learning

To model how observers update their subjective value *Q* of a target *i* at trial *t* from the sharing behavior *R*, scaled by the reward learning rate α, we apply a Rescorla-Wagner/*Q*-learning rule

$$Q_{t+1}^{i}=Q_{t}^{i}+\alpha \left(R_{t}-Q_{t}^{i}\right)$$

We tested whether reward learning depends on the valence of the prediction error by allowing for different learning rates depending on the sign of the prediction error

$$Q_{t+1}^{i}=Q_{t}^{i}+a^{+/-}\left(R_{t}-Q_{t}^{i}\right)$$

#### Action learning

To model how observed actions shape the subjective value of targets (*28*), we use an action learning rule. Observers update their action value *Q* of a target *i* at trial *t* from the observed action *A*, scaled by the action learning rate κ in the following manner

$$Q_{t+1}^{i}=Q_{t}^{i}+\kappa \left(A_{t}-Q_{t}^{i}\right)$$

We tested whether action learning occurred at the stimulus or group level by evaluating separate models, which updated either the chosen target’s or the entire group’s *Q* values.

#### Combined reward and action learning

To model how observers learned from both rewards and actions, *Q* values were updated in the following manner

$$Q_{t+1}^{i}=Q_{t}^{i}+\alpha \left(R_{t}-Q_{t}^{i}\right)+\kappa \left(A_{t}-Q_{t}^{i}\right)$$

*Q* values were converted to decision probabilities in the test phase using a standard Softmax function, in which a target’s values $Q_{t}^{i}$ were evaluated against the alternative’s values $Q_{t}^{j}$ to predict behavior on each trial. Participants’ values for *Q* were generated in the observational learning phase and subsequently fit to participants’ test phase behavior.

#### Model space

We constructed a model space from the models described above, in which reward learning occurred in either a valence-dependent or symmetrical manner, and action learning occurred at either the target or group level. Our final model space included six models: basic reward learning, valence-dependent reward learning, target-level action learning, group-level action learning, a hybrid model with valence-dependent reward learning and target-level action learning, and a hybrid model with valence-dependent reward learning and group-level action learning. Each model contained a *Β* parameter for choice stochasticity or (inverse) temperature.

#### Simulations

We simulated data from the winning hybrid model sampling from participants’ best-fitting parameters. These parameters were used to generate choice behavior on each trial, with 100 random instantiations of the experiment per participant, to prevent dependence on contingencies (e.g., trial order).

#### Parameter estimation and model comparison

Model parameters were estimated by minimizing the negative log likelihood of the model given each observer’s test phase responses, across values of the model’s free parameters [upper/lower bounds for all learning rates: (0;1), *Β*: (−100;100)]. The best-fitting parameter estimates are shown in the computational modeling section of the Supplementary Materials.
