# Supplementary material for: Transmission of social bias through observational learning
Source: Sci Adv. 2024 Jun 28;10(26):eadk2030. doi: 10.1126/sciadv.adk2030 (PMC11212708; doi:10.1126/sciadv.adk2030)
Supplement: Supplementary file 1 — Supplementary Text Figs. S1 to S12 Tables S1 to S4 [file sciadv.adk2030_sm.pdf]

Science Advances
AAAS

Supplementary Materials for

Transmission of social bias through observational learning

David T. Schultner *et al.*

Corresponding author: David T. Schultner, david.schultner@ki.se; David M. Amodio, david.amodio@gmail.com

Sci. Adv. **10**, eadk2030 (2024)
DOI: 10.1126/sciadv.adk2030

This PDF file includes:

Supplementary Text
Figs. S1 to S12
Tables S1 to S4

Supplementary Text

Study 1

Overview. In study 1, we examined whether observing prejudiced behavior leads to similar biases in the observer's own behavior. Participants (henceforth called 'observers') observed real choices and outcomes of a previous generation of participants (henceforth called 'demonstrators') who took part in a probabilistic learning task and subsequently made their own choices between previously observed options.

A previous generation of participants engaged in the following task: After reading favorable and unfavorable descriptions, similar to common prejudice messages, of two fictional social groups, participants learned how often each group member shared money with them by interacting with them in a probabilistic learning task. Both groups were on average equally likely to yield rewards, rendering group membership an uninformative and misleading cue for the aim of reward maximization.

On average, participants showed a bias in favor of the group described in favorable terms, indicating that stereotype messages influenced participants' choice behavior despite the opportunity to learn objective reward rates and equal sharing rates between groups.
A full description of the methods and results of this direct learning task is available in the supplemental information of Schultner et al. (https://psyarxiv.com/mwztc/, study 1, *24*).
In the current study, novel participants first observed choices and resulting outcomes of the first generation of participants and subsequently made their own choices among previously observed stimuli.

Method

Participants. 336 participants recruited from the New York University online subject pool received course credit for their participation, as well as a chance to win $15 for the top five performers. In this and subsequent reported studies, we excluded trials with response times under 200 ms and removed participants who failed to give valid responses to more than ¼ of trials. Furthermore, we excluded participants who failed more than 50% of catch trials during the observation phase. Applying these exclusion criteria resulted in a final sample of 290 participants in study 1 (156 women, 64 men, 1 other, 69 unreported, $M_{age} = 19.42$ years, $SD_{age} = 1.26$ years).

Procedure. Following informed consent, participants learned that they would observe interactions of previous participants with members of two social groups - Groups B and G. Participants were then shown avatars representing players from each group, where different color cues (blue vs. green shirt color and lighter vs. darker hair color) indicated group membership (Figure S1). Participants saw either all male or all female avatars, where avatar gender was randomized to control for potentially confounding gender effects.

Unbeknownst to observers, demonstrators had received the following stereotype messages with positive and negative valence about the two social groups (which were labeled Group A and Group B in the direct learning experiment):

"In the main task you will play an interactive money sharing game with people from two different groups who come from different places. For the purpose of this study, we will refer to these groups as Group A and Group B, and their members will be represented by avatars. Members of Group A live in a more affluent society, where crime is low and most people have good jobs. People from Group A are often perceived to be trustworthy, honest, and generous to others, and they are proud of their success.
Group B, by comparison, lives in a society that is economically poor, with a high rate of unemployment and serious crimes such as robbery, assault, and murder. People from Group B are often perceived to be hostile, untrustworthy, and dishonest."

These descriptions reflect common stereotypes of White and Black Americans (*1*) and led demonstrators to preferentially choose members of Group A over members of Group B in the ensuing task.

Participants furthermore read that demonstrators had previously interacted with these eight players in order to learn how often each player shared a point with them, which would subsequently be converted to bonus payments. Next, participants learned that they would observe these interactions and make choices between members of the two groups themselves later in the experiment, but, unlike the first generation of participants, they did not receive the stereotype messages.

Following the instructions, participants first, as a comprehension check, completed a classification task during which they had to sort each avatar to its corresponding group. During this task, a pseudorandom succession of 32 stimuli (each avatar appeared 4 times) appeared in the center of the screen and participants had to sort each stimulus to its corresponding group (indicated by the labels 'Group B' and 'Group G' on the top left and top right of the screen) by pressing the left or right arrow key within 5000ms. Affirmative or negative feedback indicated whether the participant sorted an avatar to its correct group on each trial.

Next, participants completed the main task which was adapted from a reinforcement learning task (*2*). This probabilistic learning task was divided into an observational learning phase of 160 trials (or fewer, depending on the number of valid demonstrator responses), during which participants observed the demonstrators' choices and resulting outcomes, and a test phase of 96 trials (or fewer, again based on the number of valid demonstrator responses), during which participants made their own choices without receiving outcome feedback to preclude further learning.

During the observational learning phase, participants observed real choices between pairs of avatars, one from each group, where reward probabilities on each trial summed to 1 (see Figure S1). Demonstrators made choices between the two avatars on each trial with the corresponding response time as recorded during the direct learning experiment and within 2000 ms, followed by outcome information ('Shared +1' or 'Shared 0'). As an attention check, 20 trials in pseudorandom order ('catch trials') were followed by a prompt asking participants to indicate which player the demonstrator chose on the previous trial.

Next, during the test phase, participants made their own choices between the stimuli they observed in the previous phase. Now participants made their own choices within 2000 ms by pressing the left or right arrow key. The test phase differed from the learning phase in that there was no outcome feedback and all possible between-group combinations of avatars were shown.

After the test phase, participants completed an explicit reward estimation task, in which they were asked to estimate how often each player shared with them (*"How many times out of a hundred did this player share with the Decider?"*) by typing a number 0-100 into a textbox. This measure was included to assess whether participants were aware of the players' reward rates. Lastly, participants were asked how often the demonstrator chose each player (*"How many times out of a hundred did the Decider choose this player?"*) and were again prompted to respond with a number 0-100. This measure was included to test whether participants were aware of behavioral preferences of the demonstrator.
Lastly, and in all following studies, participants completed a demographic questionnaire, indicating their age, race, gender, and comment on the experiment.

Results

Choice behavior. We first tested whether observers formed a group-based preference of their own after viewing choices of a demonstrator. To do so, we tested the extent to which reward probability or group membership of each stimulus influenced observers' test phase choices using multiple regression. Our approach involved using a binomial regression on trial-wise data with choice probability as the outcome variable, reward rate and group membership as predictors and random slopes for all within-subjects factors (reward probability and group membership) as well as random intercepts per subject. Here, group membership was coded as a binary variable where 1 indicated that this group was described in favorable terms and 0 indicated that this group was framed in unfavorable terms *to the demonstrator*. Sharing rates were equated between groups (test for a difference in actual sharing rates between groups: t = 0.52, df = 45079, p = 0.61).

Analyses indicated significant main effects of both reward rate (β=1.15, SE=0.16, Wald z = 7.14, p<.001) and group membership (β=0.3, SE=0.12, Wald z = 2.63, p=.008) on choice probability. This result persists when accounting for individual variations in reward rates (subjective group preference: p=.043, subjective reward: p<.001).

Association of observed and expressed bias. We tested whether participants' expressed level of group bias was related to the bias they observed in the demonstrator's actions with a linear regression. We predicted average expressed bias (formalized as the share of choices for members of the group framed in positive terms during the test phase) from the average observed bias (formalized as the observed share of choices for members of the group framed in positive terms during the learning phase). Observer group bias was predicted by demonstrator group bias (β = 0.41, SE = 0.08, *Wald* z = 5.10, p < .001, corresponding to a correlation of .29 [t = 5.10, df = 288, p < .001], Figure S2).

We asked whether observers misattributed the demonstrators group preference to a difference in target behavior. To answer this question, we combined the responses to two different post-task questionnaires:

Explicit Beliefs. We furthermore assessed whether participants' subjective perceptions of a player's reward rate were influenced by actual reward rates and group membership. To this end, we employed a mixed effects model in which we predicted explicit reward ratings for each stimulus by its actual reward probability and group membership. Results indicated that reward rate estimates were predicted by group membership (β = 3.50, *SE* = 1.43, *t* = 2.40, *p* = .015), such that participants reported higher sharing rates for members of the group framed in favorable terms. This group difference was predicted by the demonstrator's bias (β = 21.66, *SE* = 10.4, *t* = 2.08, *p* = .038), and also by the action learning parameter derived from the best-fitting model (β = 3.36, *SE* = 1.56, *t* = 2.16, *p* = .032).

Awareness of demonstrator choices. Next, we investigated whether participants' estimates of demonstrator preferences were shaped by reward rates and group membership. To this end, we regressed explicit estimates of demonstrator choices on each player's actual reward rate and group membership. Results demonstrated the presence of a reward effect: β = 31.29, *SE* = 1.83, Wald *z* = 17.10, *p* < .001), but the absence of a group effect (group effect: β = 1.70, *SE* = 1.45, Wald *z* = 1.17, *p* = .24), indicating that participants were unaware of factors that influenced the demonstrator's choices.

We followed this analysis, which yielded a nonsignificant effect, up with a Bayesian model comparison, in which we evaluated whether the group factor had indeed an effect on participants' ratings. Results yield a bayes factor of 0.047, indicating overwhelming support in favor of the null hypothesis. However, when testing the link between observed bias and explicitly reported choice bias at the subject-level, participants' ratings did indeed show a moderate correlation with the bias they observed (*r*=.45, *t* = 8, *p* < .0001). This result suggests that, while there was no average effect, participants were, on the individual level, at least somewhat aware of the demonstrators' preferences.

Simultaneous regression showed that while observers' own preferences were influenced by both their perceptions of player rewards (β = 0.11, *t* = 11.19, *p* < .001) and demonstrator preferences (β = 0.03, *t* = 3.00, *p* = .003), (mis)perceived player rewards had a stronger influence on their own choice preferences (*F* = 21.7, *p* < .0001, linear contrast of standardized beta coefficients).

Study 2

Overview. In study 2, we aimed to replicate the effect found in study 1 with a randomly drawn sample using the same pool of demonstrators as in study 1. Thus, for each new participant, we selected one demonstrator from the same pool used in study 1 at random with replacement, allowing for a sample with a different level of average group bias compared to study 1. The effect of this random draw was that the average demonstrator group bias during training phase was slightly larger than the average demonstrator bias during training phase found in study 1 (54.35% choices in favor of 'good' group in study 2, 53.98% choices in favor of 'good' group in study 1).

Method

Participants. We recruited 141 participants on Amazon Mechanical Turk (MTurk) who received $2.50 as well as a performance-based bonus (0-3$, median: 2$) as compensation for participating. After excluding participants as specified in study 1, the final sample includes 114 participants (45 women, 67 men, 2 unreported, $M_{age} = 33.54$years, SD_{age} =9.24 years).

Procedure. The procedure was equivalent to that of study 1, differing only in the manner demonstrators were sampled and that participants were only asked to indicate explicit reward ratings but not estimate demonstrator choices.

Results

Choice behavior. Using the same analysis strategy as in study 1 (binomial regression with maximal random effects structure), we replicated the effects of reward rate (β = 1.12, *SE* = 0.25, Wald *z* = 4.5, p < .001) and group (β = 0.83, *SE*=0.35, Wald *z* = 2.41, p = .016) on choice probability.

Association of observed and expressed bias. Furthermore, as expected, the degree of observed bias was associated with the degree of expressed bias (β = 0.65, *SE* = 0.15, Wald *z* = 4.42, p < .001, Figure S3), corresponding to a correlation of r = .39 between observed and expressed bias.

Explicit Beliefs. As in previous studies, we tested whether participants' subjective perceptions of the reward rates associated with each player could be explained by a player's objective reward probability and group membership. Results show that both reward rate (β = 16.5, *SE* = 3.77, Wald *z* = 4.38, p < .001) and social group (β = 3.69, *SE* = 1.19, Wald *z* = 3.09, p = .0019) were linked to explicit ratings, indicating that participants incorporated reward information as well as observed group preferences in their subjective reward estimates.

Study 3

Overview: In Studies 1 and 2, observed copied subtle biases from biased demonstrators (group preferences of 53.8% and 55%, respectively), but the correlation between demonstrator and observer behavior suggested that stronger demonstrator bias would also result in a proportional increase in observer bias. In Study 3, we empirically tested this prediction by repeating the previous study but only included high-bias demonstrators.
In this study, participants observed interactions of strongly biased demonstrators (above-median biased demonstrators with mean group preference of 63.4% (*SD* = 12.06%), compared with group preference in Study 1: 53.8%, *SD*=12,73%, Study 2: 55%, *SD* = 14.82%).

Method

Participants. We recruited 158 participants on Amazon Mechanical Turk (MTurk) who received $4 as well as a performance-based bonus (0-3$, median: 2$) as compensation for participating. After excluding participants as specified in study 1, the final sample includes 141 participants (72 women, 62 men, 7 unreported, $M_{age} = 41.72$ years, SD_{age} =12.42 years).

Procedure. The procedure was equivalent to that of studies 1 and 2, differing only in the pool of demonstrators from which we sampled.

Results

Choice behavior. As in previous studies, observers showed a reward effect (β = 1.45, *SE* = 0.24, Wald *z* = 6.13, p < .001) as well as a group effect (β = 1.45, *SE* = 0.31, Wald *z* = 4.69, p < .001). The average group effect was larger than in Study 1 (61% vs. 53% bias, t = 3.84, p < .001).

Association of observed and expressed bias. As previously, demonstrator bias predicted observer bias (β = 0.6, *SE* = 0.16, Wald *z* = 3.7, p < .001, Figure S4), corresponding to a correlation of r=.39 (p < .001) between observed and expressed bias.

Explicit Beliefs. Again, both reward rate (β = 19.159, *SE* = 3.28, Wald *z* = 5.84, p < .001) and social group (β = 6.6, *SE* = 1.00, Wald *z* = 6.4, p < .001) were linked to a group member's explicit reward rate.

Study 4

Overview. In study 4, we tested an alternative 'biased sampling' account that may explain the transmission of bias observed in previous studies. According to this account, observers acquired a group bias because they only had access to a highly selective subset of observations which was contingent on the demonstrators' choice behavior.

In study 4, we aimed to test this alternative account by showing participants in the experimental condition whether the unchosen group member *would have shared* a point, in addition to reward feedback from the chosen group member. Under the biased sampling account, the group preference should be reduced in the experimental condition.
To facilitate observers' detection of a group bias, we again sampled from a pool of highly biased demonstrators.

Method

Participants. We recruited 386 participants on Amazon Mechanical Turk (MTurk) who received $4 as well as a performance-based bonus (0-3$, median: 2$) as compensation for participating. After excluding participants as specified in study 1, the final sample includes 339 participants (176 participants in the control condition, 163 participants in the 'full feedback' condition, 136 women, 193 men, 1 other, 9 unreported, $M_{age} = 40.69$ years, SD_{age} =11.32 years).

Procedure. The procedure was equivalent to that in previous studies, but participants were assigned to either the control condition, in which they only received reward feedback from the group member chosen by the demonstrator, whereas participants in the experimental condition received reward feedback from both the chosen and the unchosen group member.

Results

Choice behavior. To test for the effect of receiving full feedback on participants' group preference, we added a Group x Condition interaction to the logistic regression (which was otherwise specified as previously). There was no Group x Condition interaction effect (β = 0.13, *SE* = 0.34, Wald *z* = 0.38, p = .70), but a group effect was present in both conditions (group effect in the partial feedback condition: β = 0.67, *SE* = 0.24, Wald *z* = 2.82, p = .004, group effect in the full feedback condition: β = 0.86, *SE* = 0.23, Wald *z* = 3.69, p < .001).

Association of observed and expressed bias. The correlation between demonstrator and observer bias was not modulated by condition (β = -0.16, *SE* = 0.21, *t* =-0.77, p = .4, Figure S5). Again, averaged across conditions, demonstrator bias predicted observer bias (β=-0.16, *SE*=0.1, *t* = 3.16, p = .0017, r = .17).

Study 5

Overview. In Study 5, we tested whether the presence of a human demonstrator modulates the social learning of bias. To this end, we compared a control condition, equivalent to Study 3, with a novel 'computer' condition, in which participants learned that the player selections were randomly determined by an algorithm (as opposed to caused by a human agent). We tested whether participants in the 'computer' condition would show a weaker bias than those in the control condition.

Method

Participants. We recruited 427 participants on Amazon Mechanical Turk (MTurk) who received $4.50 as well as a performance-based bonus (0-3$, median: 1.50$) as compensation for participating. After excluding participants as before, the final sample included 365 participants (184 participants in the control condition, 181 participants in the 'computer' condition, 152 women, 193 men, 5 other, 12 unreported, $M_{age} = 38.35$ years, SD_{age} =11.42 years).

Procedure. The procedure was identical to that of previous studies, but participants were either assigned to a control condition, in which they learned that a human demonstrator was making choices (as in Study 3), or a 'computer' condition, in which they learned that a computer algorithm was randomly selecting which player would provide feedback.

Results

Choice behavior. To test for the effect of the presence of a human demonstrator on participants' group preference, we added a Group x Condition interaction to the logistic regression (which was otherwise specified as previously). There was no Group x Condition interaction effect (β = 0.60, *SE* = 0.33, Wald *z* = 1.84, *p* = .067), but a group effect was present in both conditions (group effect in the control condition: β = 1.12, *SE* = 0.22, Wald *z* = 5.10, p < .001, group effect in the revealed condition: β = 1.77, *SE* = 0.25, Wald *z* = 7.23, p<.001, Figure S6).

Simultaneous regression revealed that observers' group preferences were more strongly driven by their misperception of target feedback than their perception of demonstrator choices, *F* =

49.65, $p < .0001$, linear contrast of standardized beta coefficients—a pattern that was not moderated by condition (see SI). demonstrator actions x condition interaction: $\beta = -0.01$, $SE = 0.02$, $t = -0.40$, $p = .69$, target rewards x condition interaction: $\beta = -0.00$, $SE = 0.02$, $t = -0.17$, $p = .87$.

Association of observed and expressed bias. The correlation between demonstrator and observer bias was not moderated by condition ($\beta = -0.07$, $SE = 0.18$, $t = -0.39$, p = .70, Figure S7). Again, averaged across conditions, demonstrator bias predicted observer bias ($\beta = 0.47$, $SE = 0.09$, $t = 5.37$, p < .001, rho = .22).

Additional Study

Prior to conducting Study 5, we conducted a similar study to investigate whether the presence of a human actor was necessary for the social learning of bias. All participants were assigned to a single condition (equivalent to the 'computer' condition in Study 5) in which they were told feedback for one player on each trial would be "revealed," without reference to a human demonstrator. Participants were yoked to demonstrators from Study 1, which included the full range of original demonstrators whose average choice bias was slightly in favor of the positively stereotyped group (as opposed to the high-bias demonstrators used in Study 5). Results showed that observer's preferences were influenced by target's actual feedback, [stats], as well as group membership, $\beta = 0.55$, $SE = 0.27$, Wald $z = 2.04$, p = .041, Figure S8 & S9, suggesting the transmission of group bias through social learning even in the absence of a human demonstrator. However, when an outlier was included in the analysis, the group effect was nonsignificant, $\beta = 0.5$, $SE = 0.27$, Wald $z = 1.83$, p = .067. Because this effect was relatively weak, it was considered inconclusive replaced in the main text with Study 5, which tested the same question using a stronger design.

Study 6

Overview. In study 6, we tested whether the demonstrator's perceived competence can modulate the degree to which observers acquire the demonstrator's choice bias. We manipulated perceived demonstrator competence before the observational learning phase and investigated whether the association between demonstrator and observer bias differed between two between-subjects conditions (high vs. low demonstrator competence).

Method

Participants. We recruited 355 participants on Amazon MTurk who received $3 and an additional $0-3 as performance-based bonus (median bonus: $2) by drawing six test phase trials at random and awarding $0.5 per correct choice (defined as choices for the option with equal or higher reward rate). Applying exclusion criteria yielded a final sample of 303 participants (149 women, 149 men, 1 other, 2 nonconforming, 2 unreported, $M_{age} = 40.76$years, $SD_{age} = 12.25$years).

Procedure. Instructions, main task and post-task questionnaires were equivalent to study 1, with one exception: After reading the instructions, participants received information about the

demonstrator's performance on an ostensibly previously administered decision-making task. In one condition, participants learned that demonstrators only gave 40% correct answers ('low ability condition') while in the other condition participants learned that demonstrators gave 80% correct answers ('high ability condition'). Additionally, participants saw one of two graphical representations of the demonstrator's relative performance to other ostensible participants (Figure S10).

Following the classification task (as described in study 1), participants were prompted to complete a manipulation check: First, participants were asked to indicate the percentage of correct answers the demonstrator obtained on a slider with values 0-100. Correct answers (40 for the low ability condition and 80 for the high ability condition, with deviations of up to +5 or -5 points considered correct) resulted in an affirmative message, while incorrect answers resulted in a repetition of the correct statement. Second, participants were asked to indicate if the demonstrator had scored higher, lower or average compared to other participants on the ostensibly administered decision-making task. Correct answers again led to an affirmative message and incorrect answers to a correction, followed by the first trial of the observation phase. We selected demonstrators for this experiment following a yoked sampling design (as in study 1). Each demonstrator was observed by two participants in each condition.

The test phase of the observational learning task was followed by, in addition to post-task questionnaires as in previous studies, a questionnaire regarding how confident they were in the demonstrator's ability (on a slider scale with numbers 0-100).

Results

Manipulation check. We compared the confidence participants reported to have regarding the demonstrators' competence after the learning task and find that the manipulation induced a difference in reported competence which persisted even after the learning task, in which the demonstrator's performance was, in fact, equivalent between conditions (scale of 0-100, mean low competence: 45.17, mean high competence: 69.87, $p < .001$).

Choice behavior. Again, reward probability had an effect on choice probability (β = 2.35, *SE* = 0.20, Wald z = 11.58, $p < .001$) as well as group membership (β = 0.49, *SE* = 0.20, Wald z = 2.52, p = .012), but the interaction between demonstrator ability and reward rate was not significant (β = -0.24, *SE* = 0.38, Wald z = -0.62, p = .533).

Association of observed and expressed bias. To test our main prediction that observers would follow the demonstrator's bias more closely under high than under low ability, we conducted a linear regression in which we predicted expressed bias by the interaction of observed bias and demonstrator ability. Analyses demonstrated that observers were indeed more likely to show a similar group bias as the demonstrator in the high ability condition (interaction: β = -0.51, *SE* = 0.19, Wald z = -266, p = .0082). Observed and expressed biases correlated r = .28 in the low ability condition and r = .49 in the high ability condition, indicating that participants were more likely to copy observed group biases when observing an ostensibly competent demonstrator. Excluding three potential outliers and repeating the analysis shows that the interaction is robust to excluding these outliers, β = -0.42, *SE* = 0.20, t = -2.17, p = .030 (Figure S11).

Explicit Beliefs. As in previous studies, we tested whether explicit reward ratings were shaped by reward probabilities and group membership. In this study, both reward rates (β = 30.19, *SE* = 2.44, Wald *z* =12.37, p < .001) and group membership (β = 2.43, *SE* = 0.77, Wald *z* = 3.15, p = .0016) were linked to differences in explicit reward ratings.

Computational Models

We employed reinforcement learning modeling to evaluate the contribution of observed actions and observed rewards to participants' behavior. To do so, we compared models from three different families: 1. Models that explain behavior as guided by observed reward outcomes, 2. Models that explain behavior as guided by observed actions and 3. Hybrid models that explain behavior as guided by both reward-related as well as action-related components.

Reward learning. Reward learning. To model how observers update their subjective value Q of a target $i$at trial t from the sharing behavior R, scaled by the reward learning rate α, we apply a Rescorla-Wagner/Q-learning rule:

$$Q_{t+1}^{i} = Q_{t}^{i} + \alpha(R_t - Q_t^i)$$

We tested whether reward learning depends on the valence of the prediction error by allowing for different different learning rates depending on the sign of the prediction error:

$$Q_{t+1}^{i} = Q_{t}^{i} + a^{+/-}(R_t - Q_t^i)$$

Action learning. To model how observed actions shape the subjective value of targets (*28)*, we employ an action learning rule. Observers update their action value Q of a target i at trial t from the observed action A, scaled by the action learning rate κ in the following manner:

$$Q_{t+1}^{i} = Q_{t}^{i} + \kappa(A_t - Q_t^i)$$

We tested whether action learning occurred at the stimulus or group level by evaluating separate models, which either updated the chosen target's or the entire group's Q-values.

Combined reward and action learning. To model how observers learned from both rewards and actions, Q-values were updated in the following manner:

$$Q_{t+1}^{i} = Q_{t}^{i} + \alpha(R_t - Q_t^i) \ + \kappa(A_t - Q_t^i)$$

Q-values were converted to decision probabilities in the Test phase using a standard Softmax function, in which a target's values Q_t^i were evaluated against the alternative's values Q_t^j to predict behavior on each trial:

$$P_i = \frac{e^{Q_i/\beta}}{\sum_{j=1}^{2} \ e^{Q_j/\beta}}$$

where β (0.01 < $\beta \leq$ 100) is the temperature parameter that determines the sensitivity of choices to the difference in Q-values. Very low values of β result in selecting the action with higher Q-value with probability ~1, while high values of β result in explorative choices that are insensitive to the difference in Q-values.

Participants' values for Q were generated in the observational learning phase and subsequently fit to participants' test phase behavior.

We also considered a model with separate β for positive and negative prediction errors. This model is an extension of model 5 and has five parameters: action learning, as well as positive and negative reward learning rates, and valence-dependent temperature parameters. Thus, in the observational learning phase and after experiencing a positive prediction error, the next decision was evaluated using β^{pos} and after a negative prediction error the next decision was evaluated using β^{neg}. In the test phase, there was no further reward feedback. For this case, we used the average of β^{pos} and β^{neg} as the temperature parameter.

We evaluated this model against the winning model 5 using Study 1 data and found that it provides an inferior fit (mean AIC = 121 vs. model 5 mean AIC = 115). Since this model was not a priori motivated, we did not evaluate it in the following studies.

Model fitting procedure

We used the Nelder-mead procedure in R to optimize our models. Optimization means finding a model's parameter values which, for each participant, minimize the discrepancy between predicted choice probabilities and actual choices. The Nelder-mead procedure uses a downhill simplex to arrive at the best fitting parameter values, i.e., it first performs a coarse search over the parameter space and then engages in increasingly fine searches around parameter values that provide better fits in coarser searches. Because this procedure is prone to being stuck in local optima, we repeated the search for each participant with 50 randomly chosen starting points.

Model recovery

To ensure that the computational models were identifiable, we performed model recovery analyses. This procedure involves simulating data from each computational model and then fitting each computational model on each simulation. If the model from which a dataset was simulated best fits the simulated dataset, the models are likely identifiable.

We tested this by generating simulated choices for 290 synthetic participants per model, drawing from parameter values that best fit Study 1 participants, resulting in six simulated datasets. Subsequently, we fitted all six models to all six simulated datasets, and compared the model fit per simulation using Akaike weights. Akaike weights are a transformation of the Akaike Information Criterion (AIC) and measure the relative confidence of different models being the correct model.

Figure S12. Shows the results, displayed in a confusion matrix. The diagonal values are

consistently the largest, suggesting that each model simulation was best explained by its underlying model. This confirms that all models were identifiable.

Parameter recovery

To validate that the model parameters were reliable, we performed parameter recovery. This involves comparing the parameter estimates obtained by fitting models on the simulated data, as described above, with the parameter estimates obtained by fitting models on the empirical data.

We compared these values using spearman correlations, due to the parameter estimates' non-normal distributions. The positive learning rates correlate rho = .54, S = 1859688, $p < .001$, the negative learning rates correlate rho = .50, S = 2027460, $p < .001$, the action learning rates correlate rho = .67, S = 135480, $p < .001$, and the betas correlate rho = .61, S = 1603642, $p < .001$. These consistently moderate- to strong correlation coefficients indicate that parameter recovery was reliable.

Preregistrations

Study 1 and Studies 4-6 were preregistered.

- Study 1 was preregistered at https://aspredicted.org/blind.php?x=6zi6fz. The Study 1 preregistration includes the central prediction: Observers will develop a group preference in line with the demonstrator's preference.
- Study 2 was not preregistered, but it was a nearly direct replication of Study 1 that tested the same prediction.
- Study 3 was a close replication of Study 1 that again tested the same hypothesis.
- Study 4 was preregistered at https://aspredicted.org/HQG_BC7. This preregistration included an exploratory hypothesis regarding whether a full feedback condition would differ from the partial feedback condition.
- Study 5 was preregistered at https://aspredicted.org/Y9P_W12
- The additional study was preregistered at https://aspredicted.org/G85_RCG.
- Study 6 was preregistered at https://aspredicted.org/VMI_YSV.

We applied the same exclusion criteria to all studies to achieve consistency and thus systematically deviated from the preregistered exclusion criteria. Applying the original exclusion criteria does not qualitatively change the main results.

Fig. S1.

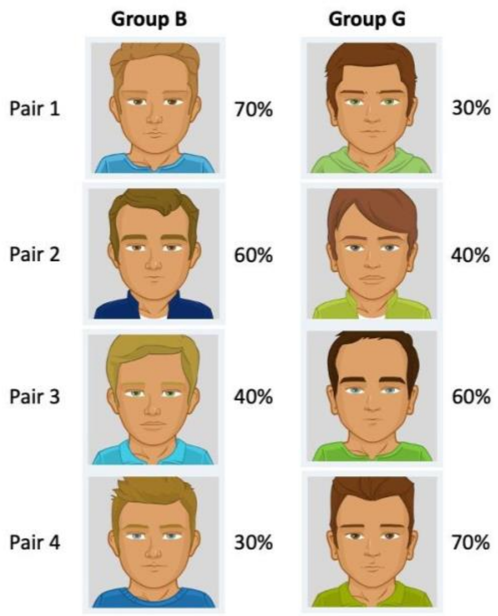

Male avatars with examples of linked reward probabilities. On each learning phase trial, one pair of avatars was shown. Image-to-reward probability mappings were randomized within groups for each participant to eliminate a potentially confounding effect of stimulus preferences.

Fig. S2

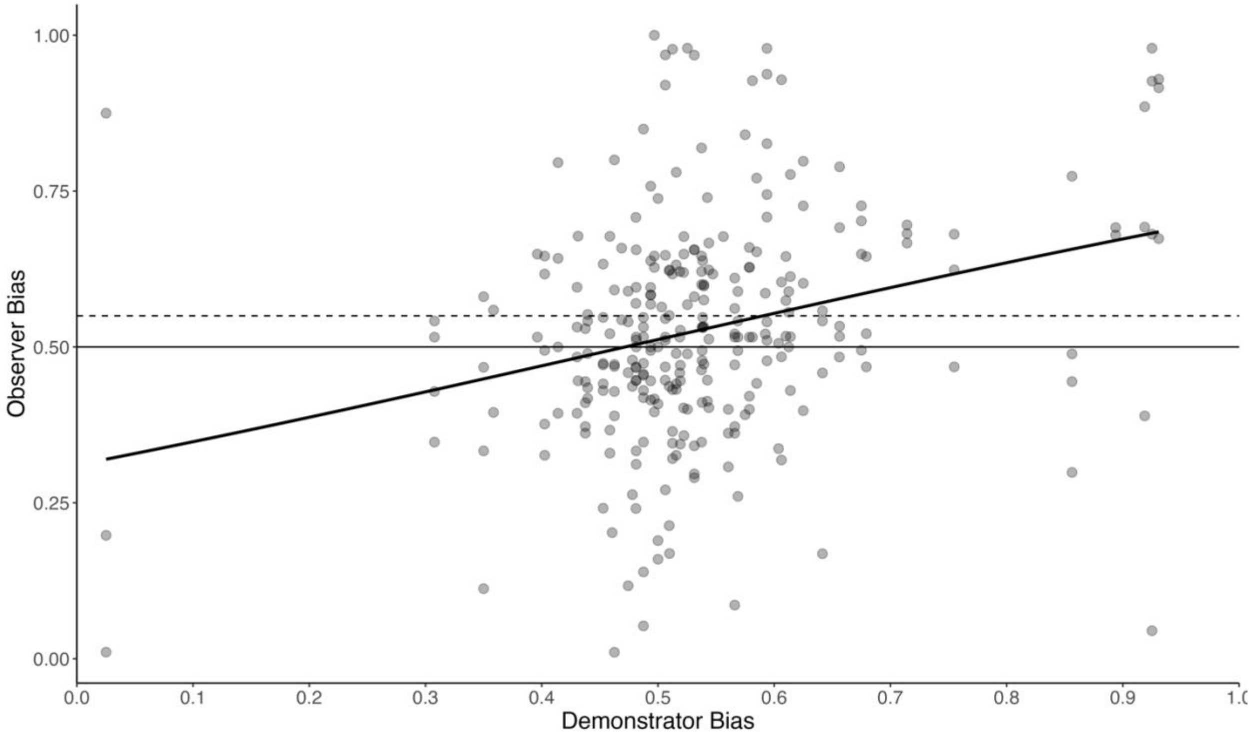

Study 1: Correlation between demonstrator and observer bias

Fig. S3

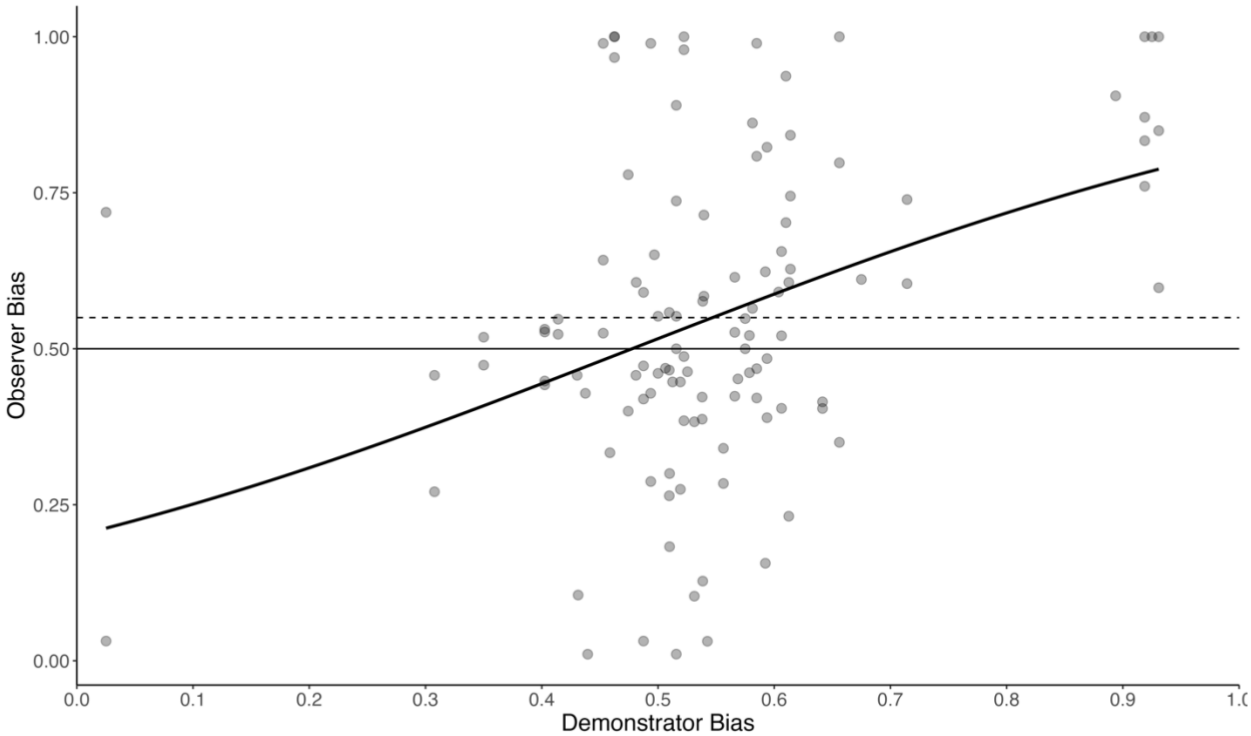

Study 2: Correlation between demonstrator and observer bias

Fig. S4

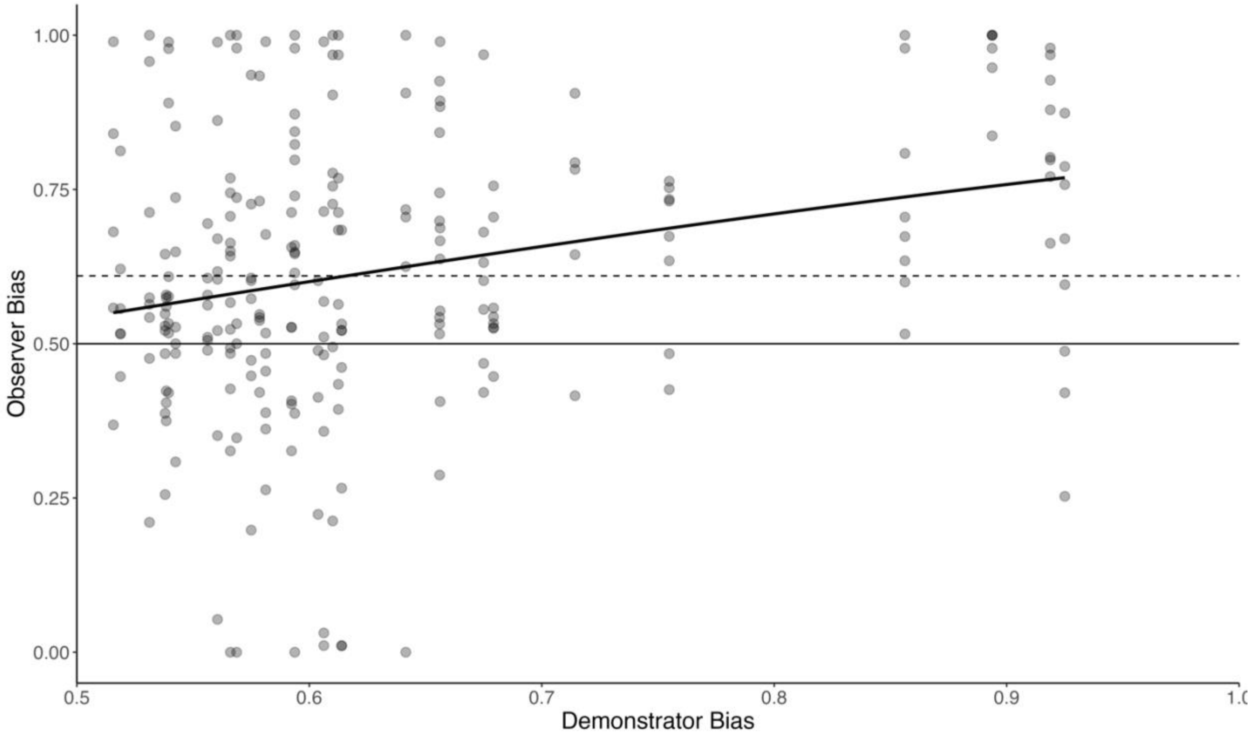

Study 3: Correlation between demonstrator and observer bias

Fig. S5

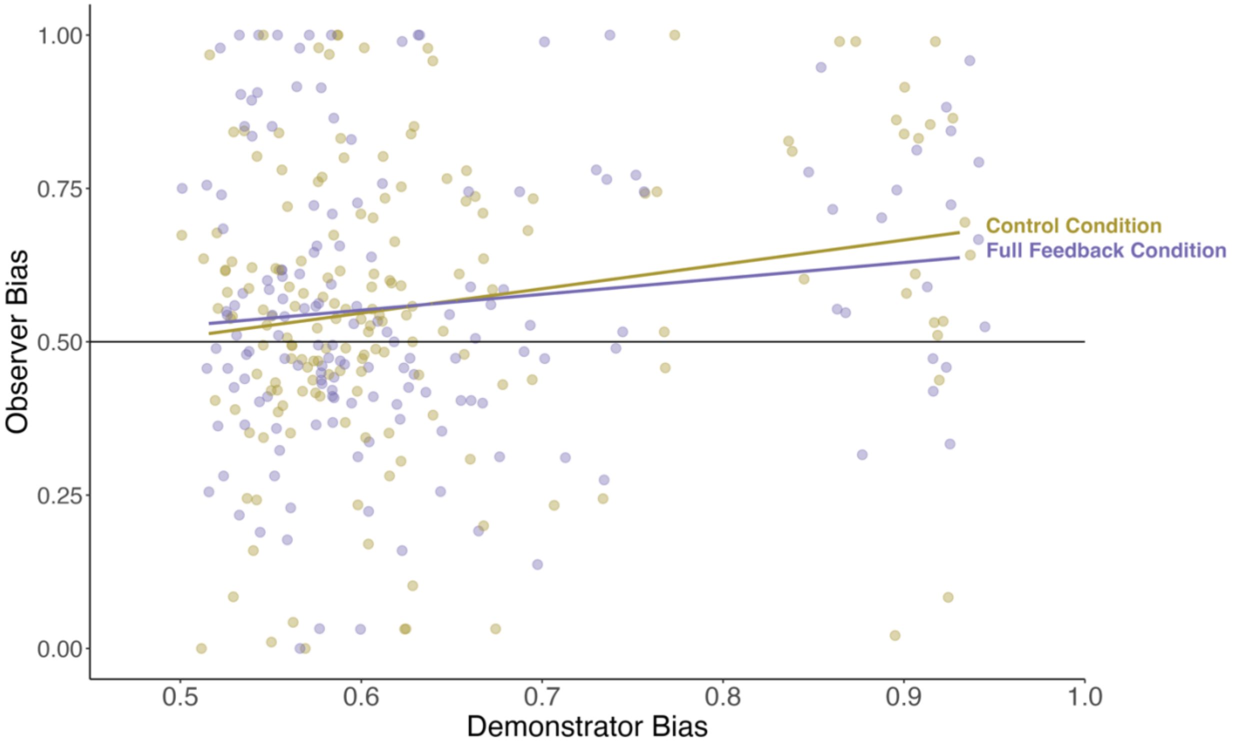

Study 4: Correlation between demonstrator and observer bias, per condition.

Fig S6.

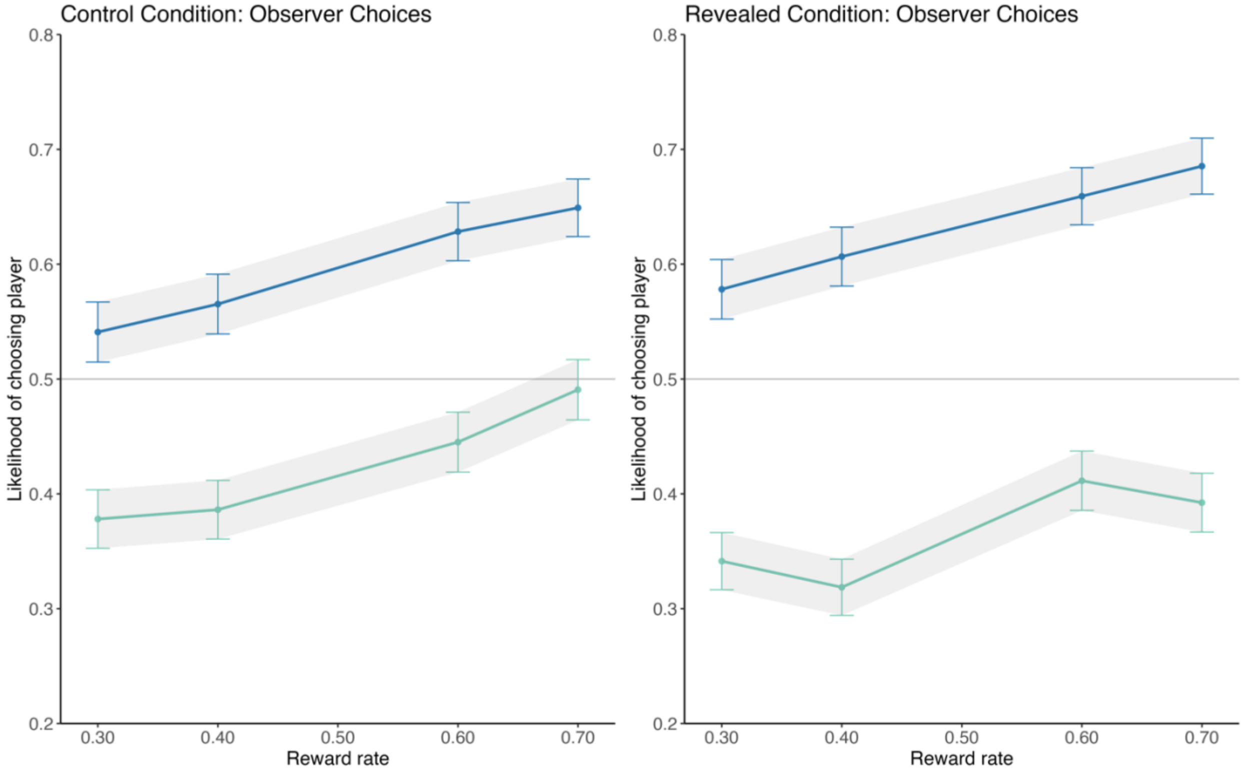

Observer choices in Study 5, displayed separately for the control condition and the revealed condition.

Fig S7.

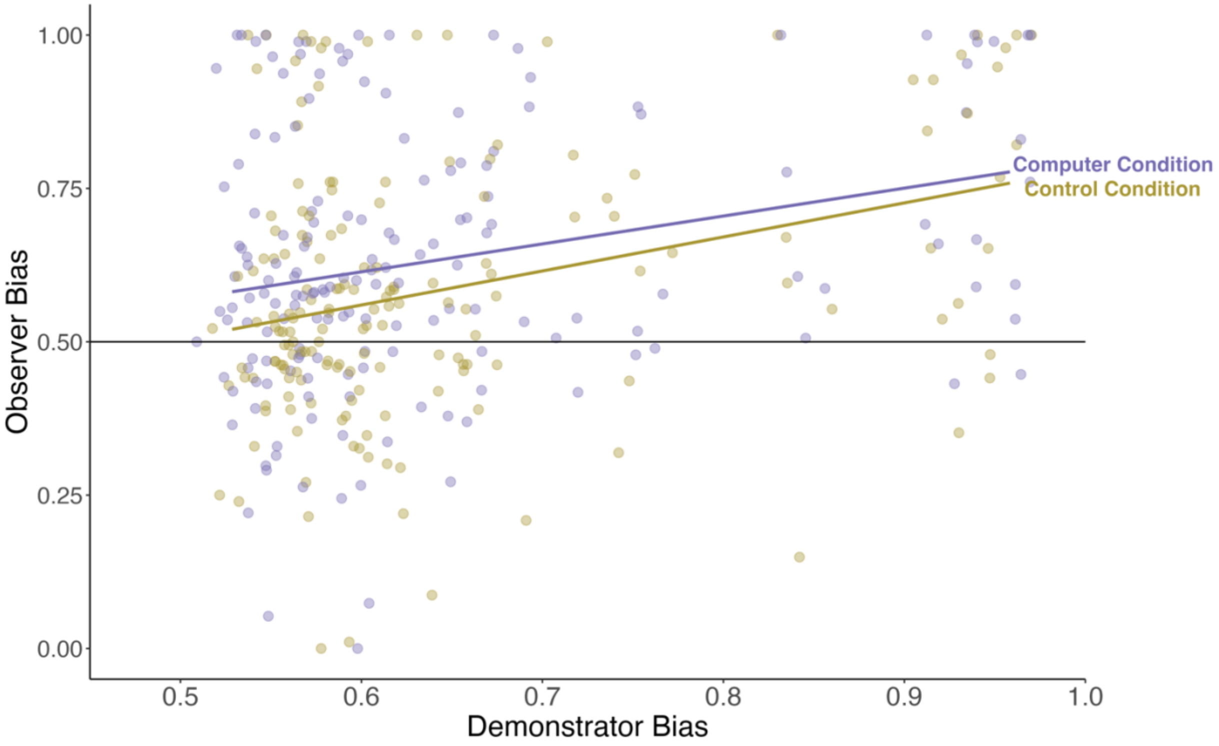

Study 5: Correlation between demonstrator and observer bias, per condition.

Fig S8.

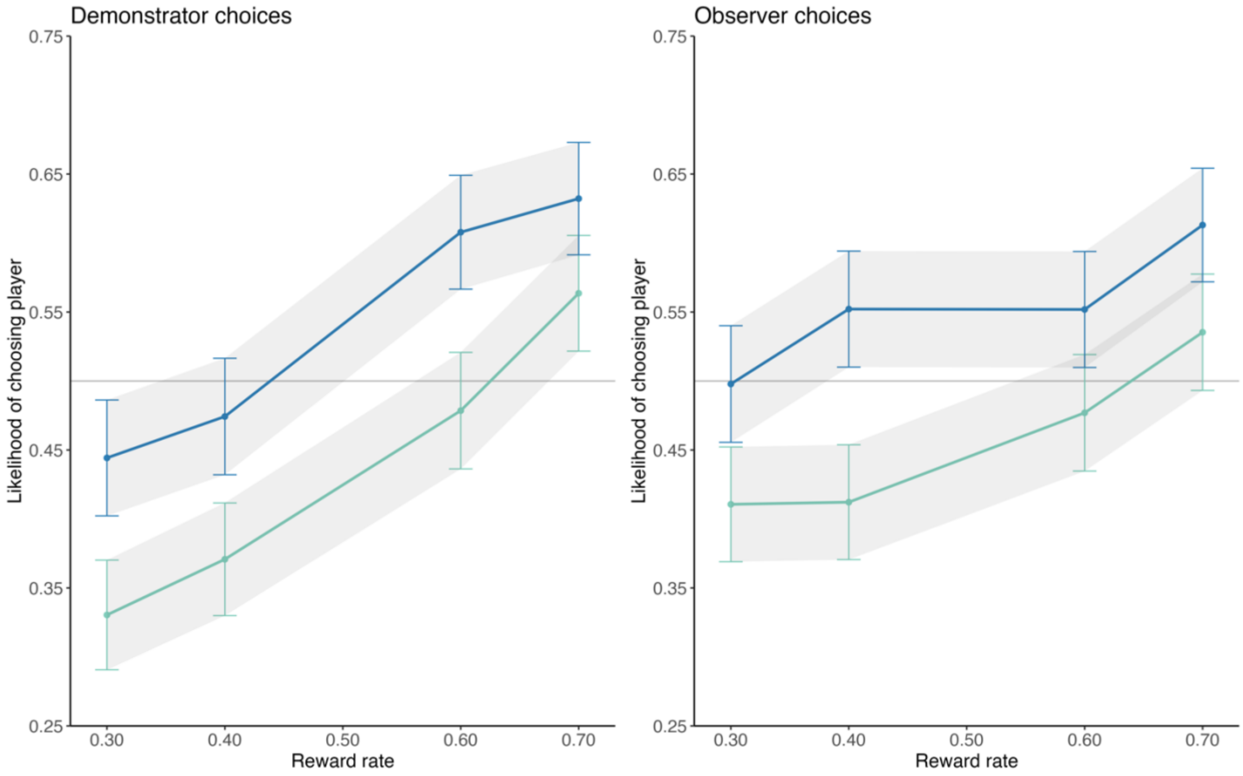

Demonstrators' and observers' choice behavior in the additional study, as part of Study 5.

Fig S9.

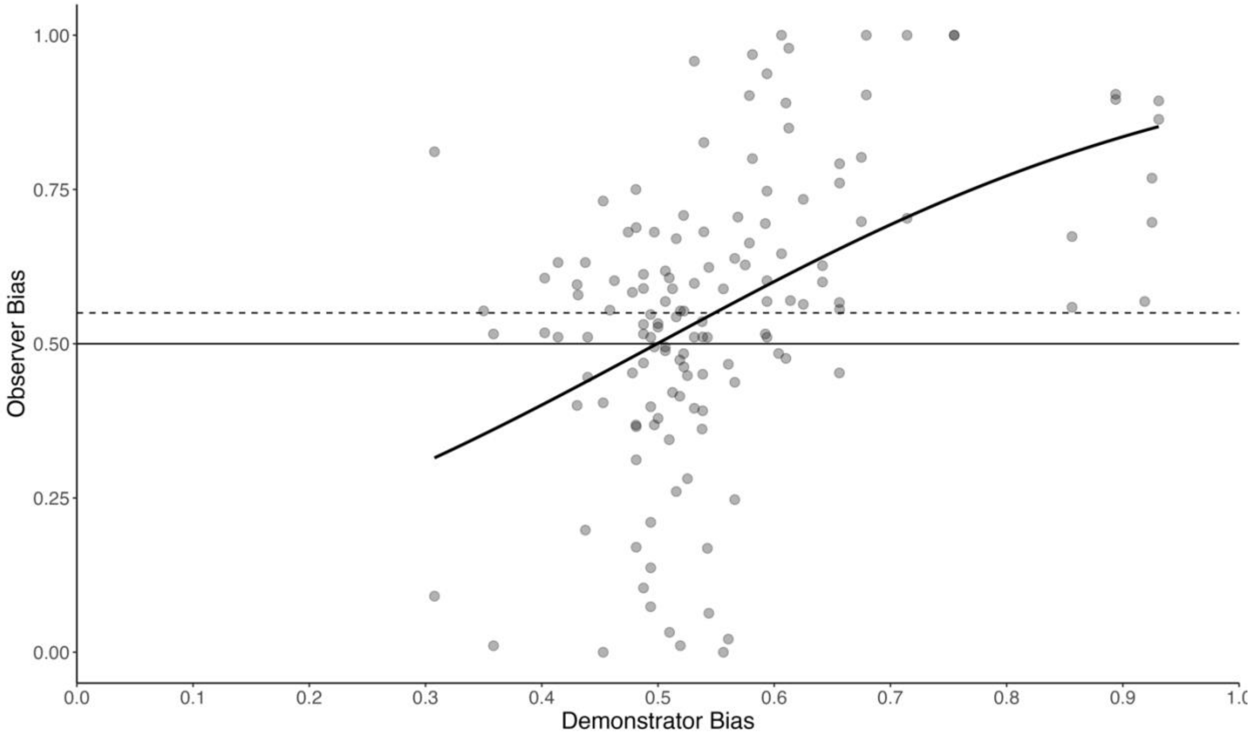

Correlation between demonstrator and observer bias in the additional study, as part of Study 5. Each data point indicates one participant. The dotted horizontal line indicates the average group preference.

Fig. S10.

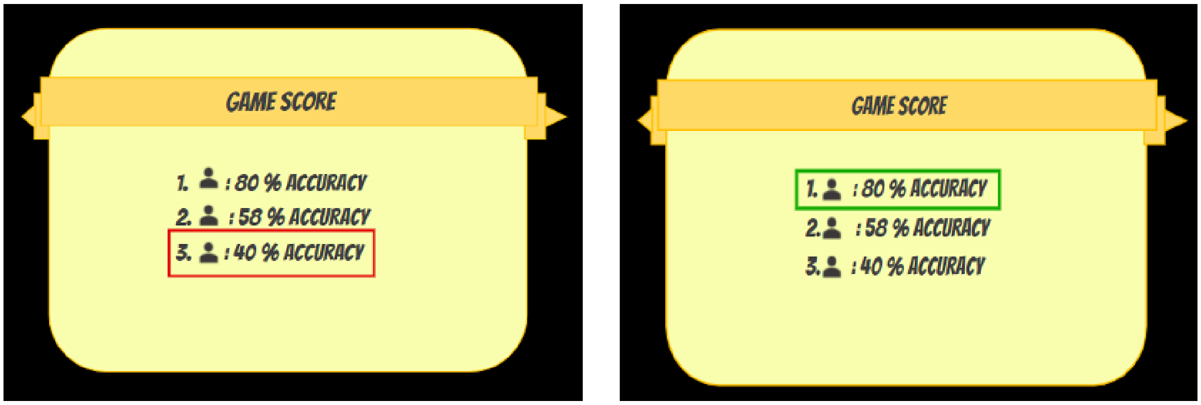

Study 6: Manipulation slides observed by participants in the low ability (left panel) and high ability condition (right panel). Right and green squares indicate the relative position on an ostensible previous decision-making task the demonstrator participated in.

Fig. S11.

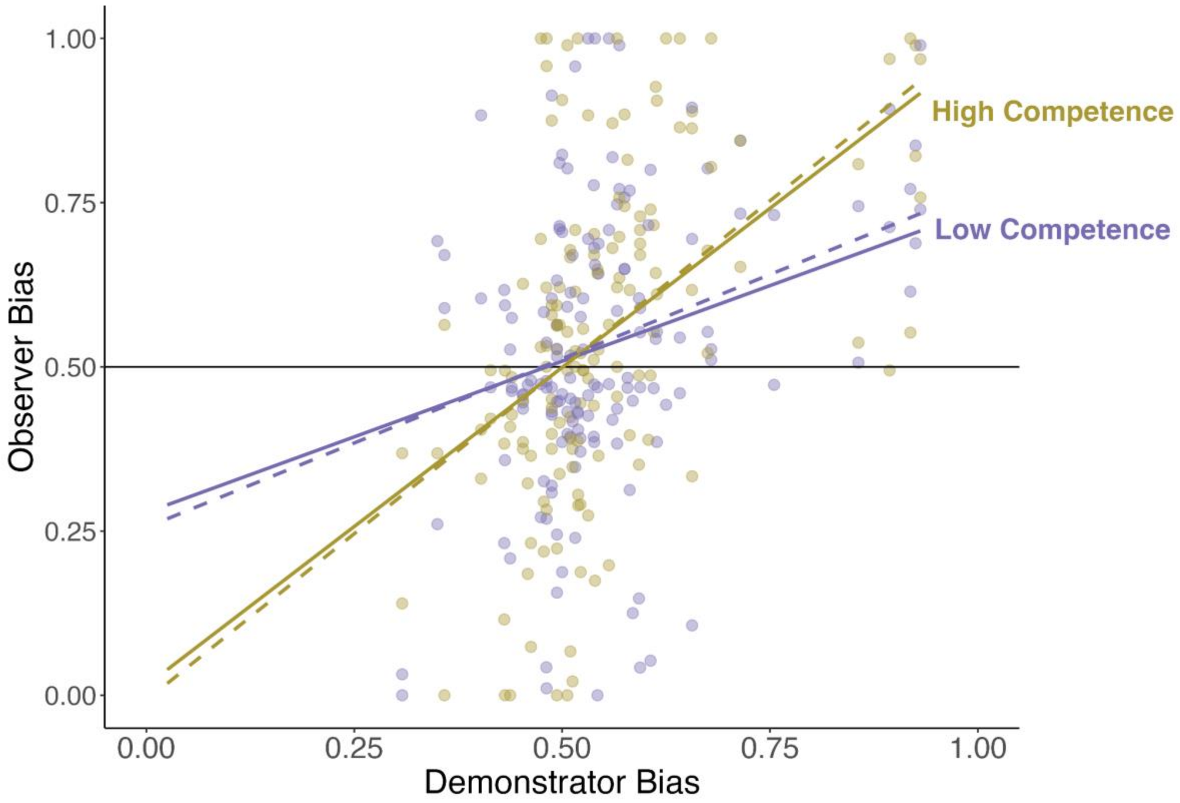

Removing outliers from the Competence X Demonstrator Bias interaction in Study 6 preserves the interaction effect.

Fig. S12.

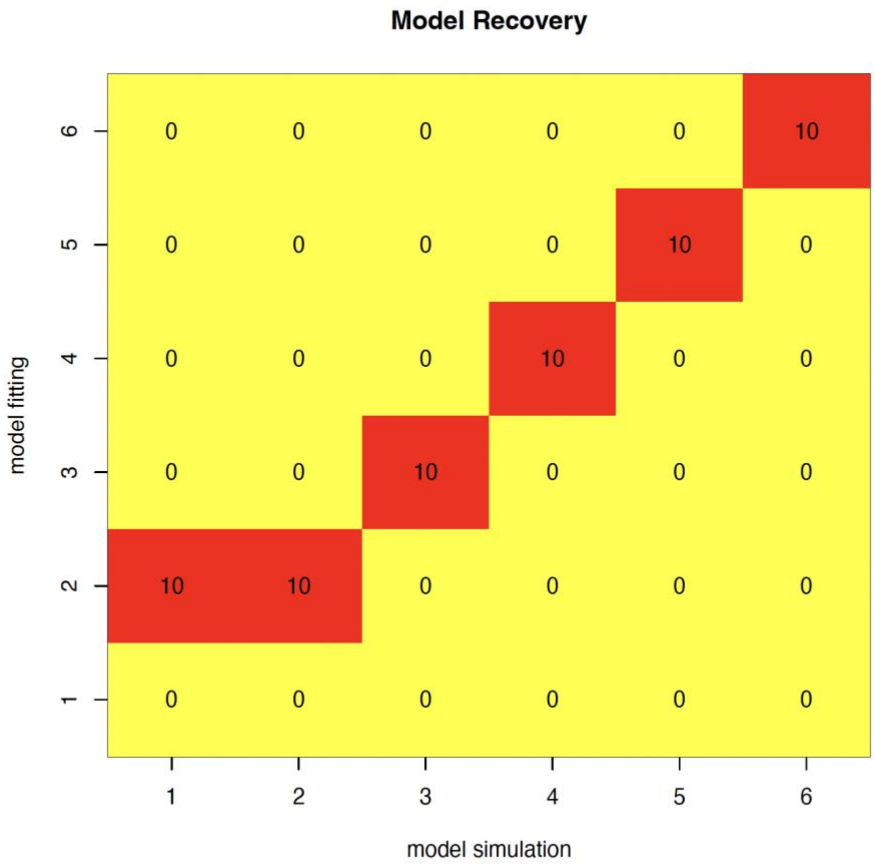

To verify that the computational models were identifiable, we simulated data from all six models of interest and fitted the data with all models. Cell values indicate the number of simulated experiments which are best fit by each model, according to mean AIC. Five out of six models, including the winning model 5, were found to be identifiable.

Table S1.

Model	label	#pars	description of parameters	model family
1	basic reward learning	2	reward learning rate {0,...,1}, beta (temperature)	reward
2	valenced reward learning	3	reward learning rate for positive and negative prediction errors {0,...,1}, beta	reward
3	group-level action learning	2	action learning rate updating for chosen group {0,...,1}, beta	action
4	target-level action learning	2	action learning rate updating for chosen target {0,...,1}, beta	action
5	valenced rewards + group-level action learning	4	reward learning rate for positive and negative prediction errors {0,...,1}, action learning rate updating for chosen group {0,...,1}, beta	hybrid
6	valenced rewards + target-level action learning	4	reward learning rate for positive and negative prediction errors {0,...,1}, action learning rate updating for specific target {0,...,1}, beta	hybrid

Model overview.

Table S2.

	M1	M2	M3	M4	M5	M6
**Study 1**	**123.56**	**119.4**	**122.07**	**121.76**	**117.80**	**115.17**
**Study 2**	**120.66**	**113.4**	**116.3**	**112.7**	**110.10**	**106.19**
**Study 3**	**122.84**	**110.57**	**111.5**	**105.54**	**107.27**	**99.17**
**Study 4**	**119.11**	**109.41**	**115.65**	**112.38**	**107.16**	**102.04**
**extra Study**	**120.65**	**111.82**	**113.09**	**108.47**	**106.05**	**99.31**
**Study 5**	**118.48**	**107.95**	**111.63**	**107.81**	**104.93**	**100.04**
**Study 6**	**118.29**	**109.51**	**122.63**	**133.69**	**103.85**	**97.98**

Model fit per study (mean AIC)

Table S3.

	alfa pos	alfa neg	alfa act	beta
**Study 1**	.34	.29	.13	.56
**Study 2**	.31	.31	.21	.41
**Study 3**	.26	.33	.22	.54
**Study 4**	.31	.27	.15	.33
**extra Study**	.33	.32	.20	.39
**Study 5**	.33	.25	.19	.42
**Study 6**	.31	.26	.15	.56

Mean parameters of Model 6

Table S4.

	**alfa pos**	**alfa neg**	**alfa act**	**beta**
**Study 1**	.19	.08	.02	.12
**Study 2**	.08	.06	.03	.16
**Study 3**	.05	.12	.04	.15
**Study 4**	.09	.08	.01	.17
**Extra Study**	.13	.08	.03	.11
**Study 5**	.12	.05	.02	.12
**Study 6**	.12	.05	.02	.08

Median parameters of Model 6
